# Prognostic factors for the outcomes of COVID-19 patients infected with SARS-CoV-2 Omicron and Delta variants

**DOI:** 10.1186/s12920-023-01637-1

**Published:** 2023-08-29

**Authors:** Gunadi, Mohamad Saifudin Hakim, Hendra Wibawa, Khanza Adzkia Vujira, Dyah Ayu Puspitarani, Endah Supriyati, Ika Trisnawati, Kristy Iskandar, Riat El Khair, Afiahayati, Siswanto, Yunika Puspadewi, Sri Handayani Irianingsih, Dwi Aris Agung Nugrahaningsih, Laudria Stella Eryvinka, Fadila Dyah Trie Utami, Edita Mayda Devana, Lanang Aditama, Nathania Christi Putri Kinasih, Yekti Hediningsih, Nur Rahmi Ananda, Marcellus, Eggi Arguni, Titik Nuryastuti, Tri Wibawa

**Affiliations:** 1 Pediatric Surgery Division, Department of Surgery/Genetics Working Group/Translational Research Unit, Faculty of Medicine, Public Health and Nursing, Universitas Gadjah Mada, Yogyakarta, Indonesia; 2 Department of Microbiology, Faculty of Medicine, Public Health and Nursing, Universitas Gadjah Mada, Yogyakarta, Indonesia; 3 Directorate General, and Livestock Services, Disease Investigation Center Wates (Balai Besar Veteriner Wates), Ministry of Agriculture Indonesia, Yogyakarta, Indonesia; 4 Centre for Tropical Medicine, Faculty of Medicine, Public Health and Nursing, Universitas Gadjah Mada, Yogyakarta, Indonesia; 5 Pulmonology Division, Department of Internal Medicine, Faculty of Medicine, Public Health and Nursing, Universitas Gadjah Mada/Dr. Sardjito Hospital, Yogyakarta, Indonesia; 6 Department of Child Health/Genetics Working Group, Faculty of Medicine, Public Health and Nursing, Universitas Gadjah Mada/UGM Academic Hospital, Yogyakarta, Indonesia; 7 Department of Clinical Pathology and Laboratory Medicine, Faculty of Medicine, Public Health and Nursing, Universitas Gadjah Mada/Dr. Sardjito Hospital, Yogyakarta, 55281 Indonesia; 8 Department of Computer Science and Electronics Faculty of Mathematics and Natural Sciences, Universitas Gadjah Mada, Yogyakarta, Indonesia; 9 Department of Physiology, Faculty of Medicine, Public Health and Nursing, Universitas Gadjah Mada/UGM Academic Hospital, Yogyakarta, Indonesia; 10 Department of Pharmacology and Therapy/Genetics Working Group, Faculty of Medicine, Public Health and Nursing, Universitas Gadjah Mada, Yogyakarta, Indonesia; 11 Balai Laboratorium Kesehatan dan Pengujian Alat Kesehatan, Central Java, Semarang, Indonesia; 12 Department of Child Health, Faculty of Medicine, Public Health and Nursing, Universitas Gadjah Mada/Dr. Sardjito Hospital, Yogyakarta, Indonesia

**Keywords:** Delta variant, Hospitalization, Mortality, Omicron variant, SARS-CoV-2, COVID-19 outcomes, Whole-genome sequencing

## Abstract

**Background:**

The SARS-CoV-2 Omicron variant has replaced the previously dominant Delta variant because of high transmissibility. However, studies on the impact of the Omicron variant on the severity of COVID-19 are still limited in developing countries. Our study aimed to determine the prognostic factors for the outcomes of patients infected with SARS-CoV-2 Omicron and Delta variants, including age, sex, comorbidities, and smoking.

**Methods:**

In this retrospective cross-sectional study, we involved 352 patients with COVID-19 from Yogyakarta and Central Java provinces, Indonesia, from May 2021 to February 2022, consisting of 164 males and 188 females. We included all patients with the PCR’s Ct value of less than 30 for further whole-genome sequencing.

**Results:**

Ct value and mean age of COVID-19 patients were not significantly different between both groups (*p* = 0.146 and 0.273, respectively). Patients infected with Omicron (n = 139) and Delta (n = 213) variants showed similar hospitalization (*p* = 0.396) and mortality rates (*p* = 0.565). Multivariate analysis of both groups showed that older age (≥ 65 years) had a higher risk for hospitalization (OR = 3.86 [95% CI = 1.29–11.5]; *p* = 0.015) and fatalities (OR = 3.91 [95% CI = 1.35–11.42]; *p* = 0.012). In both groups, patients with cardiovascular disease had a higher risk for hospitalization (OR = 5.36 [95% CI = 1.08–26.52]; *p* = 0.039), whereas patients with diabetes revealed a higher risk for fatalities (OR = 9.47 [95% CI = 3.23–27.01]; *p* = < 0.001).

**Conclusions:**

Our study shows that patients infected with Omicron and Delta variants reveal similar clinical outcomes, including hospitalization and mortality. Our findings further confirm that older age, cardiovascular disease, and diabetes are substantial prognostic factors for the outcomes of COVID-19 patients. Our findings imply that COVID-19 patients with older age, cardiovascular disease, or diabetes should be treated comprehensively and cautiously to prevent further morbidity and mortality. Furthermore, incomplete data on vaccination status hampered us from analyzing further its impact on hospitalization and mortality in our patients.

**Supplementary Information:**

The online version contains supplementary material available at 10.1186/s12920-023-01637-1.

## Introduction

Severe acute respiratory syndrome coronavirus 2 (SARS-CoV-2) is the causative agent of the ongoing global pandemic of Coronavirus disease 2019 (COVID-19) [1]. As RNA viruses, SARS-CoV-2 is undergoing continuous mutation and evolution, giving rise to novel variants with the selective fitness advantage [1]. These variants have different characteristics due to their unique sets of mutations, including transmission rate, immune escape, and clinical severity [1]. Variants with sufficient evidence of increased transmissibility, more severe diseases, or reduced vaccine or antiviral drug effectiveness have been designated a variant of concern (VOC) [2, 3]. Therefore, these variants continuously impact global health and the economy, with millions of people infected, hospitalized, and dead [2, 3].

The Omicron variant (B.1.1.529) was first identified in a sample collected in Botswana on November 11, 2021. However, South Africa first reported it on November 24, 2021. Only within two days, the World Health Organization classified Omicron as a variant of concern (VOC) on November 26, 2021 [4, 5]. In Indonesia, the surge of COVID-19 cases due to the Omicron variant occurred from late January until February 2022. Subsequently, the Omicron variant was the most frequently detected VOC compared to the previously dominating Delta variant [6]. SARS-CoV-2 genomic surveillance in Indonesia is continuously conducted to monitor circulating SARS-CoV-2 variants.

Because of high transmissibility, the SARS-CoV-2 Omicron variant has replaced the previous dominant variant, i.e., the Delta variant. It was responsible for the increase in the COVID-19 infectivity rate worldwide, including in Indonesia [7–9]. However, studies on the impact of the Omicron variant on the clinical severity of COVID-19 are still limited in developed countries [10–12]. Several studies in developed countries showed a lower clinical severity of Omicron-infected patients than Delta-infected patients [10–12]. Several factors have been associated with the clinical severity of infection, including vaccination status and the previous SARS-CoV-2 infection. In addition, we and others have shown that several prognostic factors have been associated with the outcomes of COVID-19 [13–17]. Therefore, our study aimed to determine the prognostic factors for the outcomes of patients infected with SARS-CoV-2 Omicron and Delta variants, including age, sex, comorbidities, and smoking. Our study thus contributes to informing the public health response against the emerging SARS-CoV-2 variants.

## Materials and methods

### Subjects

The inclusion criteria of this retrospective cross-sectional study were outpatient and hospitalized patients from Yogyakarta and Central Java provinces, Indonesia, from May 2021 to February 2022. The diagnosis of COVID-19 was defined according to PCR results for SARS-CoV-2. The PCR was performed on patients exhibiting COVID-19 symptoms or close contact with confirmed COVID-19 cases. We included all patients with the PCR’s Ct value of less than 30 for further whole-genome sequencing (WGS). Cases were excluded if the information was missing for any adjustment variable. We ascertained 352 patients, consisting of 164 males and 188 females, for final analysis. The outcomes of patients with COVID-19 were hospitalization and mortality. The Medical and Health Research Ethics Committee of the Faculty of Medicine, Public Health, and Nursing, Universitas Gadjah Mada, approved our study (KE/FK/0563/EC/2020). The consent was informed to all participants and parents or guardians. Written informed consent was obtained from all participants and parents or guardians for participating in this study. All data of medical records were fully anonymized.

### WGS of SARS-CoV-2

We collected all samples from nasopharyngeal swabs of outpatient or hospitalized patients with COVID-19 from May 2021 to February 2022. Subsequently, samples were sent to the COVID-19 Testing Laboratory Network in Yogyakarta for PCR and to Balai Besar Veteriner Wates and Balai Besar Teknik Kesehatan Lingkungan dan Pengendalian Penyakit Yogyakarta for WGS using MiSeq Illumina Platform.

We conducted WGS of SARS-CoV-2 for all samples with PCR’s Ct value of less than 30. As described in our previous studies [13,14,], single-stranded cDNA was synthesized from total RNA extracted from the samples of COVID-19 patients by SuperScript™ III First-Strand Synthesis System (Thermo Fisher Scientific, MA, United States). Subsequently, the second strand was synthesized by COVID-19 ARTIC v3 primer pool design by SARS-CoV-2 ARTIC Network using Phusion™ High-Fidelity DNA Polymerase (Thermo Fisher Scientific, MA, United States). The library preparations were performed using the Illumina DNA Prep (Illumina, California, United States). The Illumina MiSeq next-generation sequencer was used to perform the WGS of SARS-CoV-2. The genomes of our samples were assembled and mapped into the reference genome from Wuhan, China (hCoV-19/Wuhan/Hu-1/2019, GenBank accession number: NC_045512.2) using Burrow-Wheeler Aligner (BWA) algorithm embedded in UGENE v. 1.30 [18].

### Phylogenetic study

A dataset of 392 available SARS-CoV-2 genomes was extracted from GISAID from our region and others (Acknowledgment Table is provided in Supp. Table 1) to reconstruct the phylogenetic tree. This included SARS-CoV-2 genomes from 352 virus samples collected from our study in Central Java and Yogyakarta Special Regions provinces during Delta and Omicron variant infection waves in 2021 and 2022 and 40 virus genomes of other SARS-CoV-2 variants that previously circulated in Indonesia (B.1.1.7/Alpha, B.1.466.2, B.36, and B.1.470). Firstly, a multiple nucleotide sequence alignment was performed using the MAFFT program version 7 (https://mafft.cbrc.jp/alignment/server/). The neighbor-joining statistical method with 1,000 bootstrap replications [,19,20] was used to construct a phylogenetic tree from 29.409 nucleotide length of the open reading frame (ORF) of SARS-CoV-2, followed by computation of the evolutionary distances and model of the rate variation among sites by the Kimura 2-parameter method and the gamma distribution with estimated shape parameter (α) for the dataset, respectively [21]. The DAMBE version 7 [22] was utilized to calculate the estimation of the α gamma distribution, MEGA version 10 (MEGA X) [23] for phylogenetic reconstruction, and followed by tree visualization in FigTree (http://tree.bio.ed.ac.uk/software/FigTree/) to using a Newick tree output from MEGA X.

### Prognostic variables

We associated the outcomes of COVID-19 patients with the following prognostic variables: sex; age; comorbidities, including obesity, diabetes, hypertension, cardiovascular disease, and chronic kidney disease; and smoking. According to the previous reports, those prognostic factors were selected [13–17]. The prognostic factors, such as diabetes, hypertension, cardiovascular disease, and chronic kidney disease, were measured by the attending physician according to the diagnostic criteria of each variable. At the same time, the attending physician asked about the patient’s smoking status during the history taking. Vaccination status was not included in the analyses.

### Statistical analysis

The data were presented as mean ± SD and frequency (percentage). We determined the normality of the continuous variables by the Kolmogorov-Smirnov test. We excluded the missing or incomplete data from the final analysis. We used Chi-square or Fisher exact tests with a 95% confidence interval (CI) to find any significant association between variables and the outcomes of COVID-19 patients. Subsequently, all variables were included in the multivariate logistic regression analysis. The primary null hypothesis is that the patients infected with the Omicron variant have no greater risk of hospitalization or death than those infected with the Delta variant. We considered the *p*-value of < 0.05 as significant. We conducted all statistical analyses by the IBM Statistical Package for the Social Sciences (SPSS) version 23 (Chicago, USA).

## Results

### Phylogenetic study

**Fig. 1:**
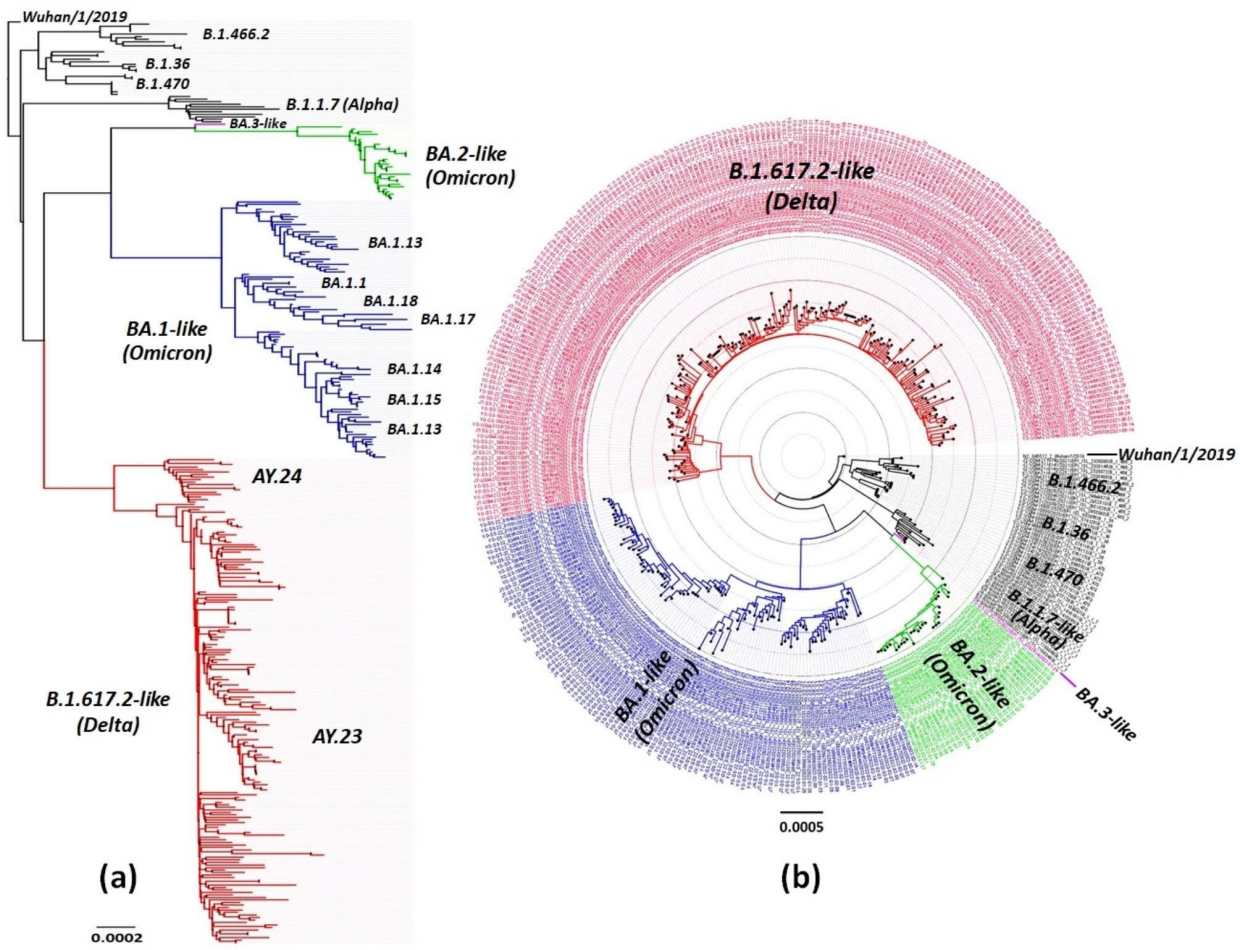
Phylogenetic analysis of Omicron and Delta variants of SARS-CoV-2 virus collected from Central Java and Yogyakarta regions from 2021–2022. Phylogenetic trees are displayed in rectangular **(a)** and polar **(b)** layouts. The evolutionary history was inferred using the Neighbor-Joining method [19, 20] and computed using the Kimura 2-parameter [21] method with 1000 bootstrap replication in MEGA X [23]. The number of base substitutions per site (0.0001) is shown on the left of the rectangular tree, where the rate variation among sites was modeled with a gamma distribution. This analysis involved 392 nucleotide sequences, a total of 29.409 positions in the final dataset. Moreover, all ambiguous positions were removed for each sequence pair (pairwise deletion option). Samples from our study were color-coded as follows: the Delta variant taxa are indicated in red, followed by Omicron-BA.1-like in blue, Omicron-BA.2-like in green, Omicron-BA.3-like in purple, and other variants previously circulating in Indonesia that are involved in this analysis are indicated in black

WGS was conducted once for each sample that met the inclusion criteria. The whole genomes generated in this study were 352. Phylogenetic analysis showed that about 60.5% (213 samples) of SARS-CoV-2 collected from Central Java and Yogyakarta provinces between May 2021 and February 2022 belonged to B.1.617.2-like (Delta variant), while 39.5% (139 samples) clustered in BA-like (Omicron variant) (Fig. 1). The majority of Delta variants were AY.23 lineage (91.5%). In contrast, a small proportion was AY.24 lineage (8.5%). For the Omicron variant, BA.1 lineage was predominantly detected (77.7%), followed by BA.2 lineage (21.6%), and only one virus (0.7%) belonged to BA.3 lineage (hcov-19/Indonesia/YO-GS-22.02175/2022: EPI_ISL_9702414). In particular, to BA.1-like virus, SARS-CoV-2 viruses from Central Java and Yogyakarta that belonged to this lineage breached into three distinct groups consisting of BA.1.13, BA.1.14, BA.1.15 (Group I), BA.1.1, BA.1.17, BA.1.18 (Group II), and a unique cluster of BA.1.13 (Group III) (Fig. 1).

### Clinical characteristics of our patients

Ct value and mean age were not significantly different between both groups (Delta: 20.36 ± 4.07 vs. Omicron: 20.5 ± 3.76; p = 0.146; and Delta: 36.53 ± 21.24 vs. Omicron: 39.06 ± 21.24; *p* = 0.273, respectively) (Table 1). All clinical characteristics of both groups were similar, except for the comorbidities of diabetes (*p* = 0.031) and chronic kidney disease (*p* = 0.017) (Table 1).

**Table 1:** Clinical characteristics of COVID-19 patients involved in this study

Characteristics	Total (N = 352) N (%); mean ± SD	Delta variant (N = 213) N (%); mean ± SD	Omicron variant (N = 139) N (%); mean ± SD	*p*-value
Ct value	20.42 ± 3.95	20.36 ± 4.07	20.5 ± 3.76	0.146
Age (years)	37.53 ± 21.23	36.53 ± 21.24	39.06 ± 21.24	0.273
≥ 65	36 (10.2%)	20 (9.4%)	16 (11.5%)	
18 - <65	235 (66.8%)	140 (65.7%)	95 (68.3%)	
< 18	81 (23%)	53 (24.9%)	28 (20.1%)	
Sex				0.072
Male	164 (46.6%)	91 (42.7%)	73 (52.5%)	
Female	188 (53.4%)	122 (57.3%)	66 (47.5%)	
Comorbidities				
Obesity	4 (1.1%)	4 (1.9%)	0 (0%)	0.156
Diabetes	29 (8.2%)	23 (10.8%)	6 (4.3%)	0.031*
Hypertension	35 (9.9%)	24 (11.3%)	11 (7.9%)	0.304
Cardiovascular disease	23 (6.5%)	12 (5.6%)	11 (7.9%)	0.398
Chronic kidney disease	7 (2%)	1 (0.5%)	6 (4.3%)	0.017*
Smoking	17 (4.8%)	12 (5.6%)	5 (3.6%)	0.384

*, significant (*p* < 0.05)

### Prognostic factors for outcomes of patients with COVID-19

The hospitalization and mortality rates were not significantly different between patients infected with Omicron and Delta variants (Table 2). Higher hospitalizations were found in older patients compared to the younger patients (OR = 7.16 [95%CI = 2.7-19.07]; *p* < 0.000), and they had a higher risk for mortality than younger ones (OR = 6.91 [95%CI = 2.84–16.86]; *p* < 0.000). Patients with diabetes, hypertension, cardiovascular disease, and chronic kidney disease showed a higher hospitalization risk. In addition, subjects with obesity, diabetes, hypertension, cardiovascular disease, and smoking revealed a higher risk for fatalities (Table 2).

**Table 2:** Prognostic factors for outcomes of COVID-19 patients

Variables	Hospitalized (N, %)	*p*-value	OR (95% CI)	Mortality (N, %)	*p*-value	OR (95% CI)
SARS-CoV-2 variant						
Delta (N = 213)	111 (52.1)	0.396	1.2	19 (8.9)	0.565	1.26
Omicron (N = 139)	66 (47.5)		(0.78–1.84)	10 (7.2)		(0.57–2.80)
Age						
18-<65 (N = 235)	109 (46.4)	Ref		14 (6)	Ref	
≥ 65 (N = 36)	31 (86.1)	0.000*	7.16	11 (30.6)	0.000*	6.94
			(2.7–19.07)			(2.84–16.93)
< 18 (N = 81)	37 (45.7)		0.97	4 (4.9)	0.733	0.82
		0.257	(0.58–1.61)			(0.26–2.56)
Sex						
Female (N = 188)	96 (51.1)	0.754	1.07	13 (6.9)	0.33	0.68
Male (N = 164)	81 (49.4)		(0.704–1.62)	16 (9.8)		(0.32–1.47)
Comorbidity						
a. Obesity						
Yes (N = 4)	4 (100)	0.123	-	2 (50)	0.035*	11.90
No (N = 348)	173 (49.9)			27 (7.8)		(1.61–87.76)
b. Diabetes						
Yes (N = 29)	29 (100)	0.000*	-	13 (44.8)	0.000*	15.59
No (N = 323)	148 (45.8)			16 (5)		(6.41–37.8)
c. Hypertension						
Yes (N = 35)	29 (80)	0.000*	4.51	11 (31.4)	0.000*	7.61
No (N = 317)	149 (47)		(1.91–10.62)	18 (5.7)		(3.22–17.95)
d. Cardiovascular disease						
Yes (N = 23)	21 (91.3)	0.000*	11.64	7 (30.4)	0.001*	6.10
No (N = 329)	156 (47.4)		(2.68–50.46)	22 (6.7)		(2.27–16.39)
e. Chronic kidney disease						
Yes (N = 7)	7 (100)	0.015*	-	1 (14.3)	0.45	1.88
No (N = 345)	170 (49.3)			28 (8.1)		(0.22–16.23)
Smoking						
Yes (N = 17)	9 (52.9)	0.832	1.11	4 (23.5)	0.042*	3.82
No (N = 335)	168 (50.1)		(0.42–2.96)	25 (7.5)		(1.16–12.57)

*, significant (*p* < 0.05); CI, confidence interval; OR, odds ratio; -, not applicable (OR is incalculable due to a divide-by-zero error)

### Multivariate analysis of prognostic factors

Subsequently, we conducted a multivariate analysis in both groups to determine the independent prognostic factors for the hospitalization and mortality of patients with COVID-19. The analysis revealed that older age (> 65 years) had a higher risk of being hospitalized (OR = 3.86 [95% CI = 1.29–11.5]; *p* = 0.015) and died (OR = 3.91 [95% CI = 1.35–11.42]; *p* = 0.012). In both groups, patients with cardiovascular disease had a higher risk of being hospitalized (OR = 5.36 [95% CI = 1.08–26.52]; *p* = 0.039), whereas patients with diabetes revealed a higher risk of mortality (OR = 9.47 [95% CI = 3.23–27.01]; *p* < 0.001) (Table 3).

**Table 3:** Multivariate analysis of prognostic factors for outcomes of patients with COVID-19.

Variables	Hospitalized		Mortality	
	OR (95% CI)	*p*-value	OR (95% CI)	*p*-value
Omicron variant (Ref: Delta variant)	1.23 (0.76–1.99)	0.382	0.88 (0.34–2.28)	0.795
Age (≥ 65 years)	3.86 (1.29–11.5)	0.015*	3.91 (1.35–11.42)	0.012*
Sex (Male)	0.94 (0.58–1.51)	0.803	1.1 (0.43–2.83)	0.82
Comorbidity				
Obesity	-	0.998	3.67 (0.32–41.72)	0.294
Diabetes	-	0.999	9.47 (3.23–27.01)	< 0.001*
Hypertension	1.79 (0.65–4.89)	0.256	2.21 (0.74–6.56)	0.153
Cardiovascular disease	5.36 (1.08–26.52)	0.039*	1.68 (0.45–6.29)	0.44
Chronic kidney disease	-	0.999	1.39 (0.09–21.63)	0.816
Smoking	0.58 (0.16–2.1)	0.41	1.96 (0.35–10.9)	0.44

*, significant (*p* < 0.05); CI, confidence interval; OR, odds ratio; -, not applicable (OR is incalculable due to a divide-by-zero error)

## Discussion

**Fig. 2:**
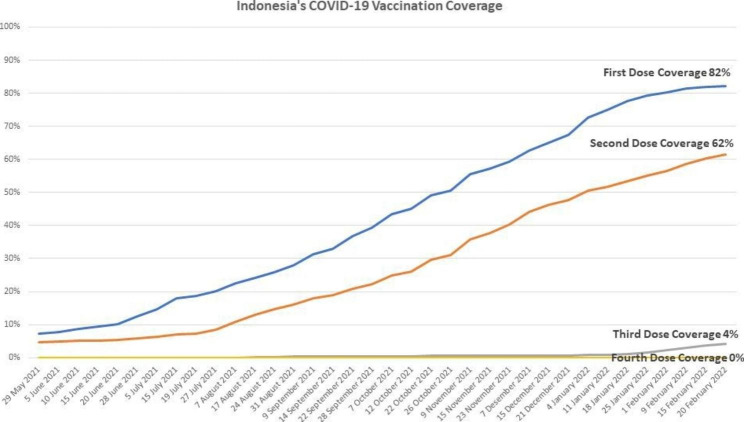
**Vaccination coverage in Indonesia during Delta and Omicron surges**

Here, we show that the outcomes of patients infected with the Omicron variant might be similar to patients infected with Delta variant regarding the hospitalization and mortality rates. Our findings were different from previous reports [10–12]. Nyberg et al. demonstrated that the outcomes of Omicron were significantly less severe than Delta and varied among ages [10]. Lewnard et al. showed that Omicron-infected patients had a lower risk of hospitalization, admission to the intensive care unit (ICU), use of ventilation, and death than Delta-infected patients. The differences were more significant in unvaccinated COVID-19 patients [11]. Importantly, Bouzid et al. indicated that Omicron patients had higher COVID-19 vaccination coverage [12]. Differences between our findings and previous reports might be affected by several variables, including host genetic background, public health measures, previous SARS-CoV-2 infections, and vaccination coverage [,24–29]. Unfortunately, incomplete data on our patients’ vaccination status and previous SARS-CoV-2 infections hampered us from further analyzing the impact of both variables on hospitalization and mortality in our patient cohort. In May 2021, the second dose vaccination coverage in Indonesia was < 10%, while in February 2022, its coverage was 62%, and already started the third dose vaccination program (Fig. 2) [30]. These differences in vaccination coverage during the Delta and Omicron surges might affect the COVID-19 patients’ outcomes. In addition, the outcome of COVID-19 varies among ethnic groups [,24–26]. Therefore, further study is essential to determine the association between host genetic risk alleles and COVID-19 outcomes in our patients. While a previous study only analyzed patients admitted to the emergency department [12], we comprehensively analyzed the patients in the hospital and community. Nevertheless, our findings might be affected by the relatively small sample size and low power of the study. The power of our study might need to be increased to detect a significant difference in the odds ratio at the magnitude reported.

Previous studies showed that older age and comorbidities are significant prognostic factors for hospitalization and mortality [13–15,, 31, 32]. Our study further provides evidence that older age, diabetes, and cardiovascular diseases as strong prognostic factors for the outcomes of COVID-19 patients. A recent systematic review showed that 50%, 20%, and 10% of older patients with COVID-19 had a severe illness, critical illness and died, respectively [33]. Several variables have attributed to the worse outcomes of COVID-19 in older patients, including associated comorbidities and delayed diagnosis due to atypical clinical manifestations [33]. 80% of older patients with COVID-19 had a minimum of one comorbidity, such as hypertension, diabetes, and cardiovascular diseases [33]. Diabetes patients might have an uncontrolled immune response and increased ACE-2 receptors and furin during SARS-CoV-2 infection, while the use of antihypertension causes aberrant ACE-2 receptor expression in hypertension patients [34]. Both mechanisms lead to the severe illness of COVID-19 patients [34]. A recent report revealed that COVID-19 patients had a higher risk of cardiovascular diseases one month after acute infection [35]. They strongly suggest that survival patients should be closely followed-up for cardiovascular diseases after acute COVID-19 infection [35].

Children were hospitalized more during the Omicron surge than during another variant surge [36]. The risk of hospitalization in children under four years during the Omicron surge was five times higher than during the Delta surge, particularly infants < 6 months old [37]. However, the COVID-19 severity was not affected by age [37]. Vaccinating eligible subjects, including pregnant women, family members, and their caregivers, is suggested to prevent children < 4 years from getting COVID-19 infection [37]. Interestingly, maternal antibodies developed following vaccination can undergo transplacental transfer. Indeed, younger children can receive protection [37]. Our study showed no difference in hospitalization and mortality rates between children and adults. Moreover, we grouped all pediatric populations from neonates, infants, young and older children into one group, i.e., < 18 years.

A previous study showed that the Ct value of Omicron was significantly higher than Delta, implying that higher transmission of Omicron does not necessarily associate with its viral load [38]. Their findings might be affected by the vaccination and prior infection of COVID-19 due to the vaccination coverage and infection rate being higher during the Omicron than the Delta surge [38]. However, our study found that the Ct value was not significantly different between Omicron and Delta variants.

Although, according to Lewnard et al., the clinical outcome of Omicron is less severe than Delta, it should be noted that Omicron has higher transmissibility and immune escape from previous COVID-19 infection and vaccination. These findings caused an extraordinary surge of COVID-19 globally and might affect the healthcare systems, including high absolute numbers of hospital admission and mortality rates [11]. Indeed, public health measures and vaccination are crucial to controlling COVID-19 spreading and decreasing morbidity and mortality.

Several limitations are noted in our study: the design of a retrospective study, incomplete data on vaccination and previous infection of COVID-19, data based on hospital admission but no data from the ICU admission unit and the use of mechanical ventilation, no follow-up data for patients after discharge from hospital, incomparable sample size between Delta and Omicron variants, and selection bias of subject patients. Another possible confounding variable of our findings was time on the central estimates of severity due to hospital pressures and different social mixing restrictions over time.

## Conclusions

Our study shows that patients infected with Omicron and Delta variants reveal similar outcomes, including hospitalization and mortality. Our findings further confirm that older age, cardiovascular disease, and diabetes are the strong prognostic factors for the outcomes of patients with COVID-19. Our findings imply that COVID-19 patients with older age, cardiovascular disease, or diabetes should be treated comprehensively and cautiously to prevent further morbidity and mortality. Furthermore, incomplete data on vaccination status hampered us from analyzing further its impact on hospitalization and mortality in our patients.

### Electronic supplementary material

Below is the link to the electronic supplementary material.

Supplementary Material 1

## Data Availability

(ADM) All data generated or analyzed during this study are included in the submission. The sequence and metadata are shared through GISAID (www.gisaid.org).
