# Supplementary material for: Prognostic factors for the outcomes of COVID-19 patients infected with SARS-CoV-2 Omicron and Delta variants
Source: BMC Med Genomics. 2023 Aug 29;16:205. doi: 10.1186/s12920-023-01637-1 (PMC10464379; doi:10.1186/s12920-023-01637-1)
Supplement: Supplementary file 1 — Supplementary Material 1 [file 12920_2023_1637_MOESM1_ESM.docx]

## Accession ID Originating Laboratory Submitting Laboratory Authors

EPI_ISL_2943235 BBTKLPP Yogyakarta Genetics Working Group (Pokja Genetik) Faculty of Medicine, Public Health and Nursing Universitas Gadjah Mada (FK-KMK UGM); Disease

Investigation Center Wates Ministry of Agriculture Indonesia; Department of Microbiology FK-KMK UGM; Laboratorium Diagnostik Yayasan Tahija World Mosquito Program (WMP) Yogyakarta Center for Tropical Medicine FK-KMK UGM; Integrated Research Center FK-KMK UGM; Department of Computer Science and Electronics FMIPA UGM; RSUP Dr. Sardjito

Afiahayati; Dwi AA Nugrahaningsih; Dwi Indaryati; Edwin W. Daniwijaya; Eggi Arguni; Endah Supriyati; Gunadi; Hana F Hanifin; Hendra Wibawa; Indarto Sulistiyono; Irene Tania; Kristy Iskandar; Ludhang P. Rizki; Marcellus; Mohamad S. Hakim; Nungki Anggorowati; Pramesti G Dewi; Siswanto; Titik Nuryastuti; Tri Wibawa

EPI_ISL_2964704, EPI_ISL_2964706

EPI_ISL_2964697, EPI_ISL_2964700

DINKES KAB. GROBOGAN Genetics Working Group (Pokja Genetik) Faculty of Medicine, Public Health and Nursing Universitas Gadjah Mada (FK-KMK UGM); Disease Investigation Center Wates Ministry of Agriculture Indonesia; Department of Microbiology FK-KMK UGM; Laboratorium Diagnostik Yayasan Tahija World Mosquito Program (WMP) Yogyakarta Center for Tropical Medicine FK-KMK UGM; Integrated Research Center FK-KMK UGM; Department of

Computer Science and Electronics FMIPA UGM; RSUP Dr. Sardjito

DINKES KAB. JEPARA Genetics Working Group (Pokja Genetik) Faculty of Medicine, Public Health and Nursing Universitas Gadjah Mada (FK-KMK UGM); Disease Investigation Center Wates Ministry of Agriculture Indonesia; Department of Microbiology FK-KMK UGM; Laboratorium Diagnostik Yayasan Tahija World Mosquito Program (WMP) Yogyakarta Center for Tropical Medicine FK-KMK UGM; Integrated Research Center FK-KMK UGM; Department of

Computer Science and Electronics FMIPA UGM; RSUP Dr. Sardjito

Afiahayati; Dwi AA Nugrahaningsih; Dwi Indaryati; Dyah A Puspitarani; Edwin W. Daniwijaya; Eggi Arguni; Endah Supriyati; Gunadi; Hana F Hanifin; Hendra Wibawa; Indarto Sulistiyono; Irene Tania; Kristy Iskandar; Ludhang P. Rizki; Marcellus; Mohamad S. Hakim; Nungki Anggorowati; Pramesti G Dewi; Siswanto; Susan Simanjaya; Titik Nuryastuti; Tri Wibawa

Afiahayati; Alvina A Setiawan; Cita S Amalia; Dwi AA Nugrahaningsih; Dwi Indaryati; Edwin W. Daniwijaya; Eggi Arguni; Endah Supriyati; Gunadi; Hendra Wibawa; Indarto Sulistiyono; Khanza a Vujira; Kristy Iskandar; Ludhang P. Rizki; Marcellus; Mohamad S. Hakim; Nungki Anggorowati; Pramesti G Dewi; Siswanto; Titik Nuryastuti; Tri Wibawa

EPI_ISL_2954684 DKK Gunungkidul/ Puskesmas Girisubo Genetics Working Group (Pokja Genetik) Faculty of Medicine, Public Health and Nursing Universitas Gadjah Mada (FK-KMK UGM); Disease

Investigation Center Wates Ministry of Agriculture Indonesia; Department of Microbiology FK-KMK UGM; Laboratorium Diagnostik Yayasan Tahija World Mosquito Program (WMP) Yogyakarta Center for Tropical Medicine FK-KMK UGM; Integrated Research Center FK-KMK UGM; Department of Computer Science and Electronics FMIPA UGM; RSUP Dr. Sardjito

Afiahayati; Alvina A Setiawan; Cita S Amalia; Dwi AA Nugrahaningsih; Dwi Indaryati; Edwin W. Daniwijaya; Eggi Arguni; Endah Supriyati; Gunadi; Hendra Wibawa; Indarto Sulistiyono; Kristy Iskandar; Ludhang P. Rizki; Marcellus; Mohamad S. Hakim; Nungki Anggorowati; Pramesti G Dewi; Siswanto; Titik Nuryastuti; Tri Wibawa

EPI_ISL_2955335, EPI_ISL_2955586, EPI_ISL_2955597, EPI_ISL_2955598

DKK Gunungkidul/ Puskesmas Karangmojo I Genetics Working Group (Pokja Genetik) Faculty of Medicine, Public Health and Nursing Universitas Gadjah Mada (FK-KMK UGM); Disease Investigation Center Wates Ministry of Agriculture Indonesia; Department of Microbiology FK-KMK UGM; Laboratorium Diagnostik Yayasan Tahija World Mosquito Program (WMP) Yogyakarta Center for Tropical Medicine FK-KMK UGM; Integrated Research Center FK-KMK UGM; Department of

Computer Science and Electronics FMIPA UGM; RSUP Dr. Sardjito

Afiahayati; Alvina A Setiawan; Dwi AA Nugrahaningsih; Dwi Indaryati; Dyah A Puspitarani; Edwin W. Daniwijaya; Eggi Arguni; Endah Supriyati; Gunadi; Hana F Hanifin; Hendra Wibawa; Indarto Sulistiyono; Irene Tania; Khanza a Vujira; Kristy Iskandar; Ludhang P. Rizki; Marcellus; Mohamad S. Hakim; Nungki Anggorowati; Pramesti G Dewi; Siswanto; Susan Simanjaya; Titik Nuryastuti; Tri Wibawa

EPI_ISL_2933110 DKK Sleman/Puskesmas Depok 1 Genetics Working Group (Pokja Genetik) Faculty of Medicine, Public Health and Nursing Universitas Gadjah Mada (FK-KMK UGM); Disease

Investigation Center Wates Ministry of Agriculture Indonesia; Department of Microbiology FK-KMK UGM; Laboratorium Diagnostik Yayasan Tahija World Mosquito Program (WMP) Yogyakarta Center for Tropical Medicine FK-KMK UGM; Integrated Research Center FK-KMK UGM; Department of Computer Science and

Afiahayati; Alvina A Setiawan; Dwi AA Nugrahaningsih; Dwi Indaryati; Edwin W. Daniwijaya; Eggi Arguni; Endah Supriyati; Gunadi; Hendra Wibawa; Indarto Sulistiyono; Khanza a Vujira; Kristy Iskandar; Ludhang P. Rizki; Marcellus; Mohamad S. Hakim; Nungki Anggorowati; Pramesti G Dewi; Siswanto; Titik Nuryastuti; Tri Wibawa

EPI_ISL_2932613, EPI_ISL_2933109, EPI_ISL_2943237, EPI_ISL_2955609

EPI_ISL_2955627, EPI_ISL_2955628

DKK Sleman/Puskesmas Depok 1 Genetics Working Group (Pokja Genetik) Faculty of Medicine, Public Health and Nursing Universitas Gadjah Mada (FK-KMK UGM); Disease Investigation Center Wates Ministry of Agriculture Indonesia; Department of Microbiology FK-KMK UGM; Laboratorium Diagnostik Yayasan Tahija World Mosquito Program (WMP) Yogyakarta Center for Tropical Medicine FK-KMK UGM; Integrated Research Center FK-KMK UGM; Department of

Computer Science and Electronics FMIPA UGM; RSUP Dr. Sardjito

DKK Sleman/Puskesmas Ngemplak 2 Genetics Working Group (Pokja Genetik) Faculty of Medicine, Public Health and Nursing Universitas Gadjah Mada (FK-KMK UGM); Disease Investigation Center Wates Ministry of Agriculture Indonesia; Department of Microbiology FK-KMK UGM; Laboratorium Diagnostik Yayasan Tahija World Mosquito Program (WMP) Yogyakarta Center for Tropical Medicine FK-KMK UGM; Integrated Research Center FK-KMK UGM; Department of

Computer Science and Electronics FMIPA UGM; RSUP Dr. Sardjito

Afiahayati; Alvina A Setiawan; Cita S Amalia; Dwi AA Nugrahaningsih; Dwi Indaryati; Dyah A Puspitarani; Edwin W. Daniwijaya; Eggi Arguni; Endah Supriyati; Gunadi; Hana F Hanifin; Hendra Wibawa; Indarto Sulistiyono; Irene Tania; Kristy Iskandar; Ludhang P. Rizki; Marcellus; Mohamad S. Hakim; Nungki Anggorowati; Pramesti G Dewi; Siswanto; Susan Simanjaya; Titik Nuryastuti; Tri Wibawa

Afiahayati; Dwi AA Nugrahaningsih; Dwi Indaryati; Dyah A Puspitarani; Edwin W. Daniwijaya; Eggi Arguni; Endah Supriyati; Gunadi; Hana F Hanifin; Hendra Wibawa; Indarto Sulistiyono; Irene Tania; Kristy Iskandar; Ludhang P. Rizki; Marcellus; Mohamad S. Hakim; Nungki Anggorowati; Pramesti G Dewi; Siswanto; Susan Simanjaya; Titik Nuryastuti; Tri Wibawa

EPI_ISL_2932610 KOTA Yogya DKK Gondokusuman II Genetics Working Group (Pokja Genetik) Faculty of Medicine, Public Health and Nursing Universitas Gadjah Mada (FK-KMK UGM); Disease

Investigation Center Wates Ministry of Agriculture Indonesia; Department of Microbiology FK-KMK UGM; Laboratorium Diagnostik Yayasan Tahija World Mosquito Program (WMP) Yogyakarta Center for

EPI_ISL_2964681 Puskesmas Colomadu 1 DKK Karanganyar Genetics Working Group (Pokja Genetik) Faculty of Medicine, Public Health and Nursing Universitas Gadjah Mada (FK-KMK UGM); Disease

Investigation Center Wates Ministry of Agriculture Indonesia; Department of Microbiology FK-KMK UGM; Laboratorium Diagnostik Yayasan Tahija World Mosquito Program (WMP) Yogyakarta Center for Tropical Medicine FK-KMK UGM; Integrated Research Center FK-KMK UGM; Department of Computer Science and Electronics FMIPA UGM; RSUP Dr. Sardjito

Afiahayati; Alvina A Setiawan; Dwi AA Nugrahaningsih; Dwi Indaryati; Edwin W. Daniwijaya; Eggi Arguni; Endah Supriyati; Gunadi; Hendra Wibawa; Indarto Sulistiyono; Khanza a Vujira; Kristy Iskandar; Ludhang P. Rizki; Marcellus; Mohamad S. Hakim; Nungki Anggorowati; Pramesti G Dewi; Siswanto; Titik Nuryastuti; Tri Wibawa

Afiahayati; Alvina A Setiawan; Dwi AA Nugrahaningsih; Dwi Indaryati; Edwin W. Daniwijaya; Eggi Arguni; Endah Supriyati; Gunadi; Hendra Wibawa; Indarto Sulistiyono; Khanza a Vujira; Kristy Iskandar; Ludhang P. Rizki; Marcellus; Mohamad S. Hakim; Nungki Anggorowati; Pramesti G Dewi; Siswanto; Titik Nuryastuti; Tri Wibawa

EPI_ISL_2964917, EPI_ISL_2964918, EPI_ISL_2964919, EPI_ISL_2964920, EPI_ISL_2964921

EPI_ISL_2964707, EPI_ISL_2964872

RS Dr. OEN KANDANG SAPI SOLO Genetics Working Group (Pokja Genetik) Faculty of Medicine, Public Health and Nursing Universitas Gadjah Mada (FK-KMK UGM); Disease Investigation Center Wates Ministry of Agriculture Indonesia; Department of Microbiology FK-KMK UGM; Laboratorium Diagnostik Yayasan Tahija World Mosquito Program (WMP) Yogyakarta Center for Tropical Medicine FK-KMK UGM; Integrated Research Center FK-KMK UGM; Department of

Computer Science and Electronics FMIPA UGM; RSUP Dr. Sardjito

RS Dr. Oen Kandang Sapi Solo Genetics Working Group (Pokja Genetik) Faculty of Medicine, Public Health and Nursing Universitas Gadjah Mada (FK-KMK UGM); Disease Investigation Center Wates Ministry of Agriculture Indonesia; Department of Microbiology FK-KMK UGM; Laboratorium Diagnostik Yayasan Tahija World Mosquito Program (WMP) Yogyakarta Center for Tropical Medicine FK-KMK UGM; Integrated Research Center FK-KMK UGM; Department of

Computer Science and Electronics FMIPA UGM; RSUP Dr. Sardjito

Afiahayati; Alvina A Setiawan; Cita S Amalia; Dwi AA Nugrahaningsih; Dwi Indaryati; Dyah A Puspitarani; Edwin W. Daniwijaya; Eggi Arguni; Endah Supriyati; Gunadi; Hana F Hanifin; Hendra Wibawa; Indarto Sulistiyono; Irene Tania; Khanza a Vujira; Kristy Iskandar; Ludhang P. Rizki; Marcellus; Mohamad S. Hakim; Nungki Anggorowati; Pramesti G Dewi; Siswanto; Susan Simanjaya; Titik Nuryastuti; Tri Wibawa

Afiahayati; Alvina A Setiawan; Cita S Amalia; Dwi AA Nugrahaningsih; Dwi Indaryati; Dyah A Puspitarani; Edwin W. Daniwijaya; Eggi Arguni; Endah Supriyati; Gunadi; Hendra Wibawa; Indarto Sulistiyono; Kristy Iskandar; Ludhang P. Rizki; Marcellus; Mohamad S. Hakim; Nungki Anggorowati; Pramesti G Dewi; Siswanto; Susan Simanjaya; Titik Nuryastuti; Tri Wibawa

EPI_ISL_2955608 RSA UGM Genetics Working Group (Pokja Genetik) Faculty of Medicine, Public Health and Nursing Universitas Gadjah Mada (FK-KMK UGM); Disease

Investigation Center Wates Ministry of Agriculture Indonesia; Department of Microbiology FK-KMK UGM; Laboratorium Diagnostik Yayasan Tahija World Mosquito Program (WMP) Yogyakarta Center for Tropical Medicine FK-KMK UGM; Integrated Research Center FK-KMK UGM; Department of Computer Science and Electronics FMIPA UGM; RSUP Dr. Sardjito

EPI_ISL_2964684, EPI_ISL_2964688, EPI_ISL_2964692, EPI_ISL_2964941, EPI_ISL_2964942, EPI_ISL_2964943, EPI_ISL_2964944, EPI_ISL_2964956, EPI_ISL_2964957

see above RSUD DR. MOEWARDI Genetics Working Group (Pokja Genetik) Faculty of Medicine, Public Health and Nursing Universitas Gadjah Mada (FK-KMK UGM); Disease

Investigation Center Wates Ministry of Agriculture Indonesia; Department of Microbiology FK-KMK UGM; Laboratorium Diagnostik Yayasan Tahija World Mosquito Program (WMP) Yogyakarta Center for Tropical Medicine FK-KMK UGM; Integrated Research Center FK-KMK UGM; Department of Computer Science and Electronics FMIPA UGM; RSUP Dr. Sardjito

EPI_ISL_2964677 RSUD DR. MOEWARDI Surakarta Genetics Working Group (Pokja Genetik) Faculty of Medicine, Public Health and Nursing Universitas Gadjah Mada (FK-KMK UGM); Disease

Investigation Center Wates Ministry of Agriculture Indonesia; Department of Microbiology FK-KMK UGM; Laboratorium Diagnostik Yayasan Tahija World Mosquito Program (WMP) Yogyakarta Center for Tropical Medicine FK-KMK UGM; Integrated Research Center FK-KMK UGM; Department of Computer Science and Electronics FMIPA UGM; RSUP Dr. Sardjito

Afiahayati; Dwi AA Nugrahaningsih; Dwi Indaryati; Dyah A Puspitarani; Edwin W. Daniwijaya; Eggi Arguni; Endah Supriyati; Gunadi; Hendra Wibawa; Indarto Sulistiyono; Kristy Iskandar; Ludhang P. Rizki; Marcellus; Mohamad S. Hakim; Nungki Anggorowati; Pramesti G Dewi; Siswanto; Susan Simanjaya; Titik Nuryastuti; Tri Wibawa

Afiahayati; Alvina A Setiawan; Cita S Amalia; Dwi AA Nugrahaningsih; Dwi Indaryati; Dyah A Puspitarani; Edwin W. Daniwijaya; Eggi Arguni; Endah Supriyati; Gunadi; Hana F Hanifin; Hendra Wibawa; I Putu Aditio A; Indarto Sulistiyono; Irene Tania; Kristy Iskandar; Ludhang P. Rizki; Marcellus; Mohamad S. Hakim; Nungki Anggorowati; Pramesti G Dewi; Siswanto; Susan Simanjaya; Titik Nuryastuti; Tri Wibawa

Afiahayati; Alvina A Setiawan; Dwi AA Nugrahaningsih; Dwi Indaryati; Edwin W. Daniwijaya; Eggi Arguni; Endah Supriyati; Gunadi; Hendra Wibawa; Indarto Sulistiyono; Khanza a Vujira; Kristy Iskandar; Ludhang P. Rizki; Marcellus; Mohamad S. Hakim; Nungki Anggorowati; Pramesti G Dewi; Siswanto; Titik Nuryastuti; Tri Wibawa

EPI_ISL_2954064 RSUD Panembahan Senopati, Bantul Genetics Working Group (Pokja Genetik) Faculty of Medicine, Public Health and Nursing Universitas Gadjah Mada (FK-KMK UGM); Disease

Investigation Center Wates Ministry of Agriculture Indonesia; Department of Microbiology FK-KMK UGM; Laboratorium Diagnostik Yayasan Tahija World Mosquito Program (WMP) Yogyakarta Center for Tropical Medicine FK-KMK UGM; Integrated Research Center FK-KMK UGM; Department of Computer

Afiahayati; Dwi AA Nugrahaningsih; Dwi Indaryati; Edwin W. Daniwijaya; Eggi Arguni; Endah Supriyati; Gunadi; Hana F Hanifin; Hendra Wibawa; Indarto Sulistiyono; Irene Tania; Kristy Iskandar; Ludhang P. Rizki; Marcellus; Mohamad S. Hakim; Nungki Anggorowati; Pramesti G Dewi; Siswanto; Titik Nuryastuti; Tri Wibawa

EPI_ISL_2502616, EPI_ISL_2502627, EPI_ISL_2502628, EPI_ISL_2502629, EPI_ISL_2502667, EPI_ISL_2502687, EPI_ISL_2502717, EPI_ISL_2502763, EPI_ISL_2502764, EPI_ISL_2502765, EPI_ISL_2506039, EPI_ISL_2506040, EPI_ISL_2506041, EPI_ISL_2534078, EPI_ISL_2534079, EPI_ISL_2534082, EPI_ISL_2534289, EPI_ISL_2534338, EPI_ISL_2534346, EPI_ISL_2534376, EPI_ISL_2534377, EPI_ISL_2534379, EPI_ISL_2534454, EPI_ISL_2534456, EPI_ISL_2534457, EPI_ISL_2534458, EPI_ISL_2534490, EPI_ISL_2534518

see above RSUD dr. LOEKMONO HADI Genetics Working Group (Pokja Genetik) Faculty of Medicine, Public Health and Nursing Universitas Gadjah Mada (FK-KMK UGM); Disease

Investigation Center Wates Ministry of Agriculture Indonesia; Department of Microbiology FK-KMK UGM; Laboratorium Diagnostik Yayasan Tahija World Mosquito Program (WMP) Yogyakarta Center for Tropical Medicine FK-KMK UGM; Integrated Research Center FK-KMK UGM; Department of Computer Science and Electronics FMIPA UGM; RSUP Dr. Sardjito

EPI_ISL_2955607 RSUP Dr Sardjito Genetics Working Group (Pokja Genetik) Faculty of Medicine, Public Health and Nursing Universitas Gadjah Mada (FK-KMK UGM); Disease

Investigation Center Wates Ministry of Agriculture Indonesia; Department of Microbiology FK-KMK UGM; Laboratorium Diagnostik Yayasan Tahija World Mosquito Program (WMP) Yogyakarta Center for Tropical Medicine FK-KMK UGM; Integrated Research Center FK-KMK UGM; Department of Computer Science and Electronics FMIPA UGM; RSUP Dr. Sardjito

EPI_ISL_2943173 RSUP Dr. Sardjito Genetics Working Group (Pokja Genetik) Faculty of Medicine, Public Health and Nursing Universitas Gadjah Mada (FK-KMK UGM); Disease

Investigation Center Wates Ministry of Agriculture Indonesia; Department of Microbiology FK-KMK UGM; Laboratorium Diagnostik Yayasan Tahija World Mosquito Program

EPI_ISL_2955606 RSUP Sardjito Genetics Working Group (Pokja Genetik) Faculty of Medicine, Public Health and Nursing Universitas Gadjah Mada (FK-KMK UGM); Disease

Investigation Center Wates Ministry of Agriculture Indonesia; Department of Microbiology FK-KMK UGM; Laboratorium Diagnostik Yayasan Tahija World Mosquito Program (WMP) Yogyakarta Center for Tropical Medicine FK-KMK UGM; Integrated Research Center FK-KMK UGM; Department of Computer Science and Electronics FMIPA UGM; RSUP Dr. Sardjito

Afiahayati; Alvina A Setiawan; Cita S Amalia; Dwi AA Nugrahaningsih; Dwi Indaryati; Dyah A Puspitarani; Edwin W. Daniwijaya; Eggi Arguni; Endah Supriyati; Gunadi; Hana F Hanifin; Hendra Wibawa; Indarto Sulistiyono; Irene Tania; Khanza a Vujira; Kristy Iskandar; Ludhang P. Rizki; Marcellus; Mohamad S. Hakim; Nungki Anggorowati; Pramesti G Dewi; Siswanto; Susan Simanjaya; Titik Nuryastuti; Tri Wibawa

. Marcellus; Afiahayati; Bambang Sigit Riyanto; Dwi AA Nugrahaningsih; Edwin W. Daniwijaya; Eggi Arguni; Eko Budiono; Endah Supriyati; Gunadi; Hana F Hanifin; Hendra Wibawa; Heni Retnowulan; Ika Trisnawati; Ira Puspitawati; Irene Tania; Kristy Iskandar; Ludhang P. Rizki; Mohamad S. Hakim; Munawar Gani; Nungki Anggorowati; Nur Imma Fatimah Harahap; Nur Rahmi Ananda; Osman Sianipar; Riat El Khair; Satria Maulana; Siswanto; Sumardi; Titik Nuryastuti; Tri Wibawa; Umi Solekhah Intansari; Yunika Puspadewi; Elizabeth Henny Herningtiyas

. Marcellus; Afiahayati; Bambang Sigit Riyanto; Dwi AA Nugrahaningsih; Edwin W. Daniwijaya; Eggi Arguni; Eko Budiono; Endah Supriyati; Gunadi; Hana F Hanifin; Hendra Wibawa; Heni Retnowulan; Ika Trisnawati; Ira Puspitawati; Irene Tania; Kristy Iskandar; Ludhang P. Rizki; Mohamad S. Hakim; Munawar Gani; Nungki Anggorowati; Nur Imma Fatimah Harahap; Nur Rahmi Ananda; Osman Sianipar; Riat El Khair; Satria Maulana; Siswanto; Sumardi; Titik Nuryastuti; Tri Wibawa; Umi Solekhah Intansari; Yunika Puspadewi; Elizabeth Henny Herningtiyas

. Marcellus; Afiahayati; Alvina A Setiawan; Bambang Sigit Riyanto; Dwi AA Nugrahaningsih; Edwin W. Daniwijaya; Eggi Arguni; Eko Budiono; Endah Supriyati; Gunadi; Hendra Wibawa; Heni Retnowulan; Ika Trisnawati; Ira Puspitawati; Khanza a Vujira; Kristy Iskandar; Ludhang P. Rizki; Mohamad S. Hakim; Munawar Gani; Nungki Anggorowati; Nur Imma Fatimah Harahap; Nur Rahmi Ananda; Osman Sianipar; Riat El Khair; Satria Maulana; Siswanto; Sumardi; Titik Nuryastuti; Tri Wibawa; Umi Solekhah Intansari; Yunika Puspadewi; Elizabeth Henny Herningtiyas

# Acknowledgement EPI_SET Identifier: EPI_SET_20220426dx

## Accession ID Originating Laboratory Submitting Laboratory Authors

EPI_ISL_9433340, EPI_ISL_9433342, EPI_ISL_9433345, EPI_ISL_9433347, EPI_ISL_9433399, EPI_ISL_9487459

EPI_ISL_9433338, EPI_ISL_9487460

EPI_ISL_9435593, EPI_ISL_9435799

BB VET Wates/Puskesmas Kalibawang Wates Genetics Working Group (Pokja Genetik) Faculty of Medicine, Public Health and Nursing Universitas Gadjah Mada (FK-KMK UGM);

Disease Investigation Center Wates Ministry of Agriculture Indonesia; Department of Microbiology FK-KMK UGM; Laboratorium Diagnostik Yayasan Tahija World Mosquito Program (WMP) Yogyakarta Center for Tropical Medicine FK-KMK UGM; Integrated Research Center FK-KMK UGM; Department of Computer Science and Electronics FMIPA UGM; RSUP Dr. Sardjito

BB VET Wates/Puskesmas Samigaluh I Wates Genetics Working Group (Pokja Genetik) Faculty of Medicine, Public Health and Nursing Universitas Gadjah Mada (FK-KMK UGM);

Disease Investigation Center Wates Ministry of Agriculture Indonesia; Department of Microbiology FK-KMK UGM; Laboratorium Diagnostik Yayasan Tahija World Mosquito Program (WMP) Yogyakarta Center for Tropical Medicine FK-KMK UGM; Integrated Research Center FK-KMK UGM; Department of Computer Science and Electronics FMIPA UGM; RSUP Dr. Sardjito

BB VET Wates/Puskesmas Wates Genetics Working Group (Pokja Genetik) Faculty of Medicine, Public Health and Nursing Universitas Gadjah Mada (FK-KMK UGM); Disease Investigation Center Wates Ministry of Agriculture Indonesia; Department of Microbiology FK-KMK UGM; Laboratorium Diagnostik Yayasan Tahija World Mosquito Program (WMP) Yogyakarta Center for Tropical Medicine FK-KMK UGM; Integrated

Research Center FK-KMK UGM; Department of Computer Science and Electronics FMIPA UGM; RSUP Dr. Sardjito

Afiahayati; Dwi AA Nugrahaningsih; Dwi Indaryati; Dyah A Puspitarani; Edwin W. Daniwijaya; Eggi Arguni; Endah Supriyati; Fadila D T Utami; Gita C Gabriela; Gunadi; Hendra Wibawa; Ika Trisnawati; Indarto Sulistiyono; Khanza A Vujira; Kristy Iskandar; Lanang Aditama; Laudria S Eryvinka; Ludhang P. Rizki; Mohamad S. Hakim; Nungki Anggorowati; Pramesti G Dewi; Riat E Khair; Siswanto; Titik Nuryastuti; Tri Wibawa

Afiahayati; Dwi AA Nugrahaningsih; Dwi Indaryati; Dyah A Puspitarani; Edwin W. Daniwijaya; Eggi Arguni; Endah Supriyati; Fadila D T Utami; Gita C Gabriela; Gunadi; Hendra Wibawa; Ika Trisnawati; Indarto Sulistiyono; Khanza A Vujira; Kristy Iskandar; Lanang Aditama; Laudria S Eryvinka; Ludhang P. Rizki; Mohamad S. Hakim; Nungki Anggorowati; Pramesti G Dewi; Riat E Khair; Siswanto; Titik Nuryastuti; Tri Wibawa

Afiahayati; Dwi AA Nugrahaningsih; Dwi Indaryati; Dyah A Puspitarani; Edwin W. Daniwijaya; Eggi Arguni; Endah Supriyati; Gita C Gabriela; Gunadi; Hendra Wibawa; Indarto Sulistiyono; Khanza A Vujira; Kristy Iskandar; Ludhang P. Rizki; Mohamad S. Hakim; Nungki Anggorowati; Pramesti G Dewi; Siswanto; Titik Nuryastuti; Tri Wibawa

EPI_ISL_12241047 BBTKLPP Genetics Working Group (Pokja Genetik) Faculty of Medicine, Public Health and Nursing Universitas Gadjah Mada (FK-KMK UGM);

Disease Investigation Center Wates Ministry of Agriculture Indonesia; Department of Microbiology FK-KMK UGM; Laboratorium Diagnostik Yayasan Tahija World Mosquito Program (WMP) Yogyakarta Center for Tropical Medicine FK-KMK UGM; Integrated Research Center FK-KMK UGM; Department of Computer Science and Electronics FMIPA UGM; RSUP Dr. Sardjito

EPI_ISL_9487466, EPI_ISL_9487490, EPI_ISL_9487491, EPI_ISL_9570708, EPI_ISL_9570710, EPI_ISL_9570712, EPI_ISL_9570714, EPI_ISL_9570715, EPI_ISL_9570717, EPI_ISL_9570720, EPI_ISL_9570721, EPI_ISL_9570722

see above Balitbangkes Genetics Working Group (Pokja Genetik) Faculty of Medicine, Public Health and Nursing Universitas Gadjah Mada (FK-KMK UGM);

Disease Investigation Center Wates Ministry of Agriculture Indonesia; Department of Microbiology FK-KMK UGM; Laboratorium Diagnostik Yayasan Tahija World Mosquito Program (WMP) Yogyakarta Center for Tropical Medicine FK-KMK UGM; Integrated Research Center FK-KMK UGM; Department of Computer Science and Electronics FMIPA UGM; RSUP Dr. Sardjito

EPI_ISL_9433333 DKK Yogyakarta Genetics Working Group (Pokja Genetik) Faculty of Medicine, Public Health and Nursing Universitas Gadjah Mada (FK-KMK UGM);

Disease Investigation Center Wates Ministry of Agriculture Indonesia; Department of Microbiology FK-KMK UGM; Laboratorium Diagnostik Yayasan Tahija World Mosquito Program (WMP) Yogyakarta Center for Tropical Medicine FK-KMK UGM; Integrated Research Center FK-KMK UGM; Department of Computer Science and Electronics FMIPA UGM; RSUP Dr. Sardjito

Afiahayati; Dwi AA Nugrahaningsih; Dyah A Puspitarani; Edita M Devana; Edwin W. Daniwijaya; Eggi Arguni; Endah Supriyati; Esensi T Geometri; Fadila D T Utami; Gita C Gabriela; Gunadi; Hendra Wibawa; Ika Trisnawati; Khanza A Vujira; Kristy Iskandar; Ludhang P. Rizki; Mohamad S. Hakim; Nungki Anggorowati; Riat E Khair; Siswanto; Titik Nuryastuti; Tri Wibawa; Verrell Christopher

Afiahayati; Dwi AA Nugrahaningsih; Dyah A Puspitarani; Edwin W. Daniwijaya; Eggi Arguni; Endah Supriyati; Fadila D T Utami; Gita C Gabriela; Gunadi; Hendra Wibawa; Ika Trisnawati; Khanza A Vujira; Kristy Iskandar; Lanang Aditama; Laudria S Eryvinka; Ludhang P. Rizki; Mohamad S. Hakim; Nungki Anggorowati; Riat E Khair; Siswanto; Titik Nuryastuti; Tri Wibawa

Afiahayati; Dwi AA Nugrahaningsih; Dwi Indaryati; Dyah A Puspitarani; Edwin W. Daniwijaya; Eggi Arguni; Endah Supriyati; Gita Christy Gabriela; Gunadi; Hendra Wibawa; Indarto Sulistiyono; Khanza A Vujira; Kristy Iskandar; Ludhang P. Rizki; Mohamad S. Hakim; Nungki Anggorowati; Pramesti G Dewi; Siswanto; Titik Nuryastuti; Tri Wibawa

EPI_ISL_9487451, EPI_ISL_9487452, EPI_ISL_9487453, EPI_ISL_9487454

EPI_ISL_7162207, EPI_ISL_7162210

DKK Yogyakarta/HI-LAB Yogyakarta Genetics Working Group (Pokja Genetik) Faculty of Medicine, Public Health and Nursing Universitas Gadjah Mada (FK-KMK UGM); Disease Investigation Center Wates Ministry of Agriculture Indonesia; Department of Microbiology FK-KMK UGM; Laboratorium Diagnostik Yayasan Tahija World Mosquito Program (WMP) Yogyakarta Center for Tropical Medicine FK-KMK UGM; Integrated

Research Center FK-KMK UGM; Department of Computer Science and Electronics FMIPA UGM; RSUP Dr. Sardjito

Dinkes Kab Demak Genetics Working Group (Pokja Genetik) Faculty of Medicine, Public Health and Nursing Universitas Gadjah Mada (FK-KMK UGM); Disease Investigation Center Wates Ministry of Agriculture Indonesia; Department of Microbiology FK-KMK UGM; Laboratorium Diagnostik Yayasan Tahija World Mosquito Program (WMP) Yogyakarta Center for Tropical Medicine FK-KMK UGM; Integrated

Research Center FK-KMK UGM; Department of Computer Science and Electronics FMIPA UGM; RSUP Dr. Sardjito

Afiahayati; Dwi AA Nugrahaningsih; Dyah A Puspitarani; Edwin W. Daniwijaya; Eggi Arguni; Endah Supriyati; Fadila D T Utami; Gita C Gabriela; Gunadi; Hendra Wibawa; Ika Trisnawati; Khanza A Vujira; Kristy Iskandar; Lanang Aditama; Laudria S Eryvinka; Ludhang P. Rizki; Mohamad S. Hakim; Nungki Anggorowati; Riat E Khair; Siswanto; Titik Nuryastuti; Tri Wibawa

Afiahayati; Dwi AA Nugrahaningsih; Dwi Indaryati; Dyah A Puspitarani; Edwin W. Daniwijaya; Eggi Arguni; Endah Supriyati; Gita C Gabriela; Gunadi; Hendra Wibawa; Indarto Sulistiyono; Khanza A Vujira; Kristy Iskandar; Ludhang P. Rizki; Marcellus; Mohamad S. Hakim; Nungki Anggorowati; Pramesti G Dewi; Siswanto; Titik Nuryastuti; Tri Wibawa

EPI_ISL_10658791 Dr. Oen Hospital Genetics Working Group (Pokja Genetik) Faculty of Medicine, Public Health and Nursing Universitas Gadjah Mada (FK-KMK UGM);

Disease Investigation Center Wates Ministry of Agriculture Indonesia; Department of Microbiology FK-KMK UGM; Laboratorium Diagnostik Yayasan Tahija World Mosquito Program (WMP) Yogyakarta Center for Tropical Medicine FK-KMK UGM; Integrated Research Center FK-KMK UGM; Department of Computer Science and Electronics FMIPA UGM; RSUP Dr. Sardjito

Afiahayati; Dwi AA Nugrahaningsih; Dyah A Puspitarani; Edita M Devana; Edwin W. Daniwijaya; Eggi Arguni; Endah Supriyati; Esensi T Geometri; Fadila D T Utami; Gita C Gabriela; Gunadi; Hendra Wibawa; Ika Trisnawati; Khanza A Vujira; Kristy Iskandar; Ludhang P. Rizki; Mohamad S. Hakim; Nungki Anggorowati; Riat E Khair; Siswanto; Titik Nuryastuti; Tri Wibawa; Verrell Christopher

EPI_ISL_10657578, EPI_ISL_10657730, EPI_ISL_10658771

EPI_ISL_10657580, EPI_ISL_10657732

EPI_ISL_10639786, EPI_ISL_10657735, EPI_ISL_10658762, EPI_ISL_10658767, EPI_ISL_10658768

Dr. Oen Solo Baru Hospital Genetics Working Group (Pokja Genetik) Faculty of Medicine, Public Health and Nursing Universitas Gadjah Mada (FK-KMK UGM); Disease Investigation Center Wates Ministry of Agriculture Indonesia; Department of Microbiology FK-KMK UGM; Laboratorium Diagnostik Yayasan Tahija World Mosquito Program (WMP) Yogyakarta Center for Tropical Medicine FK-KMK UGM; Integrated

Research Center FK-KMK UGM; Department of Computer Science and Electronics FMIPA UGM; RSUP Dr. Sardjito

Hospital District. Wonosobo Genetics Working Group (Pokja Genetik) Faculty of Medicine, Public Health and Nursing Universitas Gadjah Mada (FK-KMK UGM); Disease Investigation Center Wates Ministry of Agriculture Indonesia; Department of Microbiology FK-KMK UGM; Laboratorium Diagnostik Yayasan Tahija World Mosquito Program (WMP) Yogyakarta Center for Tropical Medicine FK-KMK UGM; Integrated

Research Center FK-KMK UGM; Department of Computer Science and Electronics FMIPA UGM; RSUP Dr. Sardjito

JIH SOLO Genetics Working Group (Pokja Genetik) Faculty of Medicine, Public Health and Nursing Universitas Gadjah Mada (FK-KMK UGM); Disease Investigation Center Wates Ministry of Agriculture Indonesia; Department of Microbiology FK-KMK UGM; Laboratorium Diagnostik Yayasan Tahija World Mosquito Program (WMP) Yogyakarta Center for Tropical Medicine FK-KMK UGM; Integrated

Research Center FK-KMK UGM; Department of Computer Science and Electronics FMIPA UGM; RSUP Dr. Sardjito

Afiahayati; Dwi AA Nugrahaningsih; Dyah A Puspitarani; Edita M Devana; Edwin W. Daniwijaya; Eggi Arguni; Endah Supriyati; Esensi T Geometri; Fadila D T Utami; Gita C Gabriela; Gunadi; Hendra Wibawa; Ika Trisnawati; Khanza A Vujira; Kristy Iskandar; Ludhang P. Rizki; Mohamad S. Hakim; Nungki Anggorowati; Riat E Khair; Siswanto; Titik Nuryastuti; Tri Wibawa; Verrell Christopher

Afiahayati; Dwi AA Nugrahaningsih; Dyah A Puspitarani; Edita M Devana; Edwin W. Daniwijaya; Eggi Arguni; Endah Supriyati; Esensi T Geometri; Fadila D T Utami; Gita C Gabriela; Gunadi; Hendra Wibawa; Ika Trisnawati; Khanza A Vujira; Kristy Iskandar; Ludhang P. Rizki; Mohamad S. Hakim; Nungki Anggorowati; Riat E Khair; Siswanto; Titik Nuryastuti; Tri Wibawa; Verrell Christopher

Afiahayati; Dwi AA Nugrahaningsih; Dyah A Puspitarani; Edita M Devana; Edwin W. Daniwijaya; Eggi Arguni; Endah Supriyati; Esensi T Geometri; Fadila D T Utami; Gita C Gabriela; Gunadi; Hendra Wibawa; Ika Trisnawati; Khanza A Vujira; Kristy Iskandar; Ludhang P. Rizki; Mohamad S. Hakim; Nungki Anggorowati; Riat E Khair; Siswanto; Titik Nuryastuti; Tri Wibawa; Verrell Christopher

EPI_ISL_9702359, EPI_ISL_9702419, EPI_ISL_9702573, EPI_ISL_9702602, EPI_ISL_9702615, EPI_ISL_9713699, EPI_ISL_9713708, EPI_ISL_9713709, EPI_ISL_9713710, EPI_ISL_9713711, EPI_ISL_9713712, EPI_ISL_9753879, EPI_ISL_9754267, EPI_ISL_9754269, EPI_ISL_9754270, EPI_ISL_12241131, EPI_ISL_12241581

see above Klinik Cito DIY Genetics Working Group (Pokja Genetik) Faculty of Medicine, Public Health and Nursing Universitas Gadjah Mada (FK-KMK UGM);

Disease Investigation Center Wates Ministry of Agriculture Indonesia; Department of Microbiology FK-KMK UGM; Laboratorium Diagnostik Yayasan Tahija World Mosquito Program (WMP) Yogyakarta Center for Tropical Medicine FK-KMK UGM; Integrated Research Center FK-KMK UGM; Department of Computer Science and Electronics FMIPA UGM; RSUP Dr. Sardjito

Afiahayati; Dwi AA Nugrahaningsih; Dyah A Puspitarani; Edwin W. Daniwijaya; Eggi Arguni; Endah Supriyati; Fadila D T Utami; Gita C Gabriela; Gunadi; Hendra Wibawa; Ika Trisnawati; Khanza A Vujira; Kristy Iskandar; Lanang Aditama; Laudria S Eryvinka; Ludhang P. Rizki; Mohamad S. Hakim; Nungki Anggorowati; Riat E Khair; Siswanto; Titik Nuryastuti; Tri Wibawa

EPI_ISL_7160934, EPI_ISL_7160951, EPI_ISL_7160953, EPI_ISL_7160957, EPI_ISL_7162054, EPI_ISL_12241065

EPI_ISL_7160420, EPI_ISL_7160930, EPI_ISL_12241067, EPI_ISL_12241068

LAB PCR RSUD CILACAP Genetics Working Group (Pokja Genetik) Faculty of Medicine, Public Health and Nursing Universitas Gadjah Mada (FK-KMK UGM); Disease Investigation Center Wates Ministry of Agriculture Indonesia; Department of Microbiology FK-KMK UGM; Laboratorium Diagnostik Yayasan Tahija World Mosquito Program (WMP) Yogyakarta Center for Tropical Medicine FK-KMK UGM; Integrated

Research Center FK-KMK UGM; Department of Computer Science and Electronics FMIPA UGM; RSUP Dr. Sardjito

LAB RS Pertamina CILACAP Genetics Working Group (Pokja Genetik) Faculty of Medicine, Public Health and Nursing Universitas Gadjah Mada (FK-KMK UGM); Disease Investigation Center Wates Ministry of Agriculture Indonesia; Department of Microbiology FK-KMK UGM; Laboratorium Diagnostik Yayasan Tahija World Mosquito Program (WMP) Yogyakarta Center for Tropical Medicine FK-KMK UGM; Integrated

Research Center FK-KMK UGM; Department of Computer Science and Electronics FMIPA UGM; RSUP Dr. Sardjito

. Marcellus; Afiahayati; Alvina A Setiawan; Bambang Sigit Riyanto; Dwi AA Nugrahaningsih; Dwi Indaryati; Dyah A Puspitarani; Edwin W. Daniwijaya; Eggi Arguni; Eko Budiono; Endah Supriyati; Gita C Gabriela; Gunadi; Hendra Wibawa; Heni Retnowulan; Ika Trisnawati; Indarto Sulistiyono; Ira Puspitawati; Khanza A Vujira; Khanza a Vujira; Kristy Iskandar; Ludhang P. Rizki; Marcellus; Mohamad S. Hakim; Munawar Gani; Nungki Anggorowati; Nur Imma Fatimah Harahap; Nur Rahmi Ananda; Osman Sianipar; Pramesti G Dewi; Riat El Khair; Satria Maulana; Siswanto; Sumardi; Susan Simanjaya; Titik Nuryastuti; Tri Wibawa; Umi Solekhah Intansari; Yunika Puspadewi; Elizabeth Henny Herningtiyas

Afiahayati; Dwi AA Nugrahaningsih; Dwi Indaryati; Dyah A Puspitarani; Edwin W. Daniwijaya; Eggi Arguni; Endah Supriyati; Gita C Gabriela; Gunadi; Hendra Wibawa; Indarto Sulistiyono; Khanza A Vujira; Khanza a Vujira; Kristy Iskandar; Ludhang P. Rizki; Marcellus; Mohamad S. Hakim; Nungki Anggorowati; Pramesti G Dewi; Siswanto; Susan Simanjaya; Titik Nuryastuti; Tri Wibawa

EPI_ISL_9754617, EPI_ISL_9754619, EPI_ISL_9754620, EPI_ISL_9754765, EPI_ISL_9754857, EPI_ISL_9754978, EPI_ISL_9755096, EPI_ISL_9755169, EPI_ISL_9755171

see above Lab Intibios Genetics Working Group (Pokja Genetik) Faculty of Medicine, Public Health and Nursing Universitas Gadjah Mada (FK-KMK UGM);

Disease Investigation Center Wates Ministry of Agriculture Indonesia; Department of Microbiology FK-KMK UGM; Laboratorium Diagnostik Yayasan Tahija World Mosquito Program (WMP) Yogyakarta Center for Tropical Medicine FK-KMK UGM; Integrated Research Center FK-KMK UGM; Department of Computer Science and Electronics FMIPA UGM; RSUP Dr. Sardjito

Afiahayati; Dwi AA Nugrahaningsih; Dyah A Puspitarani; Edwin W. Daniwijaya; Eggi Arguni; Endah Supriyati; Fadila D T Utami; Gita C Gabriela; Gunadi; Hendra Wibawa; Ika Trisnawati; Khanza A Vujira; Kristy Iskandar; Lanang Aditama; Laudria S Eryvinka; Ludhang P. Rizki; Mohamad S. Hakim; Nungki Anggorowati; Riat E Khair; Siswanto; Titik Nuryastuti; Tri Wibawa

EPI_ISL_9831384, EPI_ISL_9831597, EPI_ISL_9831736

EPI_ISL_9434160, EPI_ISL_9487412

Lab Microbiology, FK-KMK UGM Genetics Working Group (Pokja Genetik) Faculty of Medicine, Public Health and Nursing Universitas Gadjah Mada (FK-KMK UGM); Disease Investigation Center Wates Ministry of Agriculture Indonesia; Department of Microbiology FK-KMK UGM; Laboratorium Diagnostik Yayasan Tahija World Mosquito Program (WMP) Yogyakarta Center for Tropical Medicine FK-KMK UGM; Integrated

Research Center FK-KMK UGM; Department of Computer Science and Electronics FMIPA UGM; RSUP Dr. Sardjito

Lab RS Bethesda Genetics Working Group (Pokja Genetik) Faculty of Medicine, Public Health and Nursing Universitas Gadjah Mada (FK-KMK UGM); Disease Investigation Center Wates Ministry of Agriculture Indonesia; Department of Microbiology FK-KMK UGM; Laboratorium Diagnostik Yayasan Tahija World Mosquito Program (WMP) Yogyakarta Center for Tropical Medicine FK-KMK UGM; Integrated

Research Center FK-KMK UGM; Department of Computer Science and Electronics FMIPA UGM; RSUP Dr. Sardjito

Afiahayati; Dwi AA Nugrahaningsih; Dyah A Puspitarani; Edwin W. Daniwijaya; Eggi Arguni; Endah Supriyati; Fadila D T Utami; Gita C Gabriela; Gunadi; Hendra Wibawa; Ika Trisnawati; Khanza A Vujira; Kristy Iskandar; Lanang Aditama; Laudria S Eryvinka; Ludhang P. Rizki; Mohamad S. Hakim; Nungki Anggorowati; Riat E Khair; Siswanto; Titik Nuryastuti; Tri Wibawa

Afiahayati; Dwi AA Nugrahaningsih; Dwi Indaryati; Dyah A Puspitarani; Edwin W. Daniwijaya; Eggi Arguni; Endah Supriyati; Fadila D T Utami; Gita C Gabriela; Gunadi; Hendra Wibawa; Ika Trisnawati; Indarto Sulistiyono; Khanza A Vujira; Kristy Iskandar; Lanang Aditama; Laudria S Eryvinka; Ludhang P. Rizki; Mohamad S. Hakim; Nungki Anggorowati; Pramesti G Dewi; Riat E Khair; Siswanto; Titik Nuryastuti; Tri Wibawa

EPI_ISL_10639785 Lab RS Ortopedi Prof. DR.R Soeharso Surakarta Genetics Working Group (Pokja Genetik) Faculty of Medicine, Public Health and Nursing Universitas Gadjah Mada (FK-KMK UGM);

Disease Investigation Center Wates Ministry of Agriculture Indonesia; Department of Microbiology FK-KMK UGM; Laboratorium Diagnostik Yayasan Tahija World Mosquito Program (WMP) Yogyakarta Center for Tropical Medicine FK-KMK UGM; Integrated Research Center FK-KMK UGM; Department of Computer Science and Electronics FMIPA UGM; RSUP Dr. Sardjito

EPI_ISL_9713703 Lab RS Panti Rapih Genetics Working Group (Pokja Genetik) Faculty of Medicine, Public Health and Nursing Universitas Gadjah Mada (FK-KMK UGM);

Disease Investigation Center Wates Ministry of Agriculture Indonesia; Department of Microbiology FK-KMK UGM; Laboratorium

Afiahayati; Dwi AA Nugrahaningsih; Dyah A Puspitarani; Edita M Devana; Edwin W. Daniwijaya; Eggi Arguni; Endah Supriyati; Esensi T Geometri; Fadila D T Utami; Gita C Gabriela; Gunadi; Hendra Wibawa; Ika Trisnawati; Khanza A Vujira; Kristy Iskandar; Ludhang P. Rizki; Mohamad S. Hakim; Nungki Anggorowati; Riat E Khair; Siswanto; Titik Nuryastuti; Tri Wibawa; Verrell Christopher

Afiahayati; Dwi AA Nugrahaningsih; Dyah A Puspitarani; Edwin W. Daniwijaya; Eggi Arguni; Endah Supriyati; Fadila D T Utami; Gita C Gabriela; Gunadi; Hendra Wibawa; Ika Trisnawati; Khanza A Vujira; Kristy Iskandar; Lanang Aditama; Laudria S Eryvinka; Ludhang P. Rizki; Mohamad S. Hakim; Nungki Anggorowati; Riat E Khair; Siswanto; Titik Nuryastuti; Tri Wibawa

EPI_ISL_7160967, EPI_ISL_7160969, EPI_ISL_7160974, EPI_ISL_7160979

Diagnostik Yayasan Tahija World Mosquito Program (WMP) Yogyakarta Center for Tropical Medicine FK-KMK UGM; Integrated Research Center FK-KMK UGM; Department of Computer Science and Electronics FMIPA UGM; RSUP Dr. Sardjito

Labkes Bantul Genetics Working Group (Pokja Genetik) Faculty of Medicine, Public Health and Nursing Universitas Gadjah Mada (FK-KMK UGM); Disease Investigation Center Wates Ministry of Agriculture Indonesia; Department of Microbiology FK-KMK UGM; Laboratorium Diagnostik Yayasan Tahija World Mosquito Program (WMP) Yogyakarta Center for Tropical Medicine FK-KMK UGM; Integrated

Research Center FK-KMK UGM; Department of Computer Science and Electronics FMIPA UGM; RSUP Dr. Sardjito

Afiahayati; Dwi AA Nugrahaningsih; Dwi Indaryati; Dyah A Puspitarani; Edwin W. Daniwijaya; Eggi Arguni; Endah Supriyati; Gita C Gabriela; Gunadi; Hendra Wibawa; Indarto Sulistiyono; Khanza A Vujira; Khanza a Vujira; Kristy Iskandar; Ludhang P. Rizki; Marcellus; Mohamad S. Hakim; Nungki Anggorowati; Pramesti G Dewi; Siswanto; Titik Nuryastuti; Tri Wibawa

EPI_ISL_7154369, EPI_ISL_7160160, EPI_ISL_7160261, EPI_ISL_7160378, EPI_ISL_9487413, EPI_ISL_9713706, EPI_ISL_9713707

see above Labkesda Bantul Genetics Working Group (Pokja Genetik) Faculty of Medicine, Public Health and Nursing Universitas Gadjah Mada (FK-KMK UGM);

Disease Investigation Center Wates Ministry of Agriculture Indonesia; Department of Microbiology FK-KMK UGM; Laboratorium Diagnostik Yayasan Tahija World Mosquito Program (WMP) Yogyakarta Center for Tropical Medicine FK-KMK UGM; Integrated Research Center FK-KMK UGM; Department of Computer Science and Electronics FMIPA UGM; RSUP Dr. Sardjito

EPI_ISL_4969011 Labkesda Bantul DIY Genetics Working Group (Pokja Genetik) Faculty of Medicine, Public Health and Nursing Universitas Gadjah Mada (FK-KMK UGM);

Disease Investigation Center Wates Ministry of Agriculture Indonesia; Department of Microbiology FK-KMK UGM; Laboratorium Diagnostik Yayasan Tahija World Mosquito Program (WMP) Yogyakarta Center for Tropical Medicine FK-KMK UGM; Integrated Research Center FK-KMK UGM; Department of Computer Science and Electronics FMIPA UGM; RSUP Dr. Sardjito

Afiahayati; Dwi AA Nugrahaningsih; Dwi Indaryati; Dyah A Purpitarani; Dyah A Puspitarani; Edwin W. Daniwijaya; Eggi Arguni; Endah Supriyati; Fadila D T Utami; Gita C Gabriela; Gunadi; Hendra Wibawa; Ika Trisnawati; Indarto Sulistiyono; Khanza A Vujira; Khanza a Vujira; Kristy Iskandar; Lanang Aditama; Lanang Aditama; Laudria S Eryvinka; Ludhang P. Rizki; Marcellus; Mohamad S. Hakim; Nungki Anggorowati; Pramesti G Dewi; Riat E Khair; Siswanto; Susan Simanjaya; Titik Nuryastuti; Tri Wibawa

Afiahayati; Alvina A Setiawan; Cita S Amalia; Dwi AA Nugrahaningsih; Dwi Indaryati; Edwin W. Daniwijaya; Eggi Arguni; Endah Supriyati; Gunadi; Hendra Wibawa; Indarto Sulistiyono; Kristy Iskandar; Ludhang P. Rizki; Marcellus; Mohamad S. Hakim; Nungki Anggorowati; Pramesti G Dewi; Siswanto; Titik Nuryastuti; Tri Wibawa

EPI_ISL_9487461, EPI_ISL_9487462, EPI_ISL_9487463

EPI_ISL_9702357, EPI_ISL_9755990, EPI_ISL_9756034

Labkesda Bantul/Puskesmas Imogiri I Bantul Genetics Working Group (Pokja Genetik) Faculty of Medicine, Public Health and Nursing Universitas Gadjah Mada (FK-KMK UGM);

Disease Investigation Center Wates Ministry of Agriculture Indonesia; Department of Microbiology FK-KMK UGM; Laboratorium Diagnostik Yayasan Tahija World Mosquito Program (WMP) Yogyakarta Center for Tropical Medicine FK-KMK UGM; Integrated Research Center FK-KMK UGM; Department of Computer Science and Electronics FMIPA UGM; RSUP Dr. Sardjito

PUSKESMAS MLATI 1 SLEMAN Genetics Working Group (Pokja Genetik) Faculty of Medicine, Public Health and Nursing Universitas Gadjah Mada (FK-KMK UGM); Disease Investigation Center Wates Ministry of Agriculture Indonesia; Department of Microbiology FK-KMK UGM; Laboratorium Diagnostik Yayasan Tahija World Mosquito Program (WMP) Yogyakarta Center for Tropical Medicine FK-KMK UGM; Integrated

Research Center FK-KMK UGM; Department of Computer Science and Electronics FMIPA UGM; RSUP Dr. Sardjito

Afiahayati; Dwi AA Nugrahaningsih; Dyah A Puspitarani; Edwin W. Daniwijaya; Eggi Arguni; Endah Supriyati; Fadila D T Utami; Gita C Gabriela; Gunadi; Hendra Wibawa; Ika Trisnawati; Khanza A Vujira; Kristy Iskandar; Lanang Aditama; Laudria S Eryvinka; Ludhang P. Rizki; Mohamad S. Hakim; Nungki Anggorowati; Riat E Khair; Siswanto; Titik Nuryastuti; Tri Wibawa

Afiahayati; Dwi AA Nugrahaningsih; Dyah A Puspitarani; Edwin W. Daniwijaya; Eggi Arguni; Endah Supriyati; Fadila D T Utami; Gita C Gabriela; Gunadi; Hendra Wibawa; Ika Trisnawati; Khanza A Vujira; Kristy Iskandar; Lanang Aditama; Laudria S Eryvinka; Ludhang P. Rizki; Mohamad S. Hakim; Nungki Anggorowati; Riat E Khair; Siswanto; Titik Nuryastuti; Tri Wibawa

EPI_ISL_9713705 PUSKESMAS Prambanan Genetics Working Group (Pokja Genetik) Faculty of Medicine, Public Health and Nursing Universitas Gadjah Mada (FK-KMK UGM);

Disease Investigation Center Wates Ministry of Agriculture Indonesia; Department of Microbiology FK-KMK UGM; Laboratorium Diagnostik Yayasan Tahija World Mosquito Program (WMP) Yogyakarta Center for Tropical Medicine FK-KMK UGM; Integrated Research Center FK-KMK UGM; Department of Computer Science and Electronics FMIPA UGM; RSUP Dr. Sardjito

Afiahayati; Dwi AA Nugrahaningsih; Dyah A Puspitarani; Edwin W. Daniwijaya; Eggi Arguni; Endah Supriyati; Fadila D T Utami; Gita C Gabriela; Gunadi; Hendra Wibawa; Ika Trisnawati; Khanza A Vujira; Kristy Iskandar; Lanang Aditama; Laudria S Eryvinka; Ludhang P. Rizki; Mohamad S. Hakim; Nungki Anggorowati; Riat E Khair; Siswanto; Titik Nuryastuti; Tri Wibawa

EPI_ISL_9755818, EPI_ISL_9755829

Puskesmas Cangkringan Genetics Working Group (Pokja Genetik) Faculty of Medicine, Public Health and Nursing Universitas Gadjah Mada (FK-KMK UGM); Disease Investigation Center Wates Ministry of Agriculture Indonesia; Department of Microbiology FK-KMK UGM; Laboratorium Diagnostik Yayasan Tahija World Mosquito Program (WMP) Yogyakarta Center for Tropical Medicine FK-KMK UGM; Integrated

Research Center FK-KMK UGM; Department of Computer Science and Electronics FMIPA UGM; RSUP Dr. Sardjito

Afiahayati; Dwi AA Nugrahaningsih; Dyah A Puspitarani; Edwin W. Daniwijaya; Eggi Arguni; Endah Supriyati; Fadila D T Utami; Gita C Gabriela; Gunadi; Hendra Wibawa; Ika Trisnawati; Khanza A Vujira; Kristy Iskandar; Lanang Aditama; Laudria S Eryvinka; Ludhang P. Rizki; Mohamad S. Hakim; Nungki Anggorowati; Riat E Khair; Siswanto; Titik Nuryastuti; Tri Wibawa

EPI_ISL_4969122, EPI_ISL_4969127, EPI_ISL_4969136, EPI_ISL_4969298, EPI_ISL_4969302, EPI_ISL_5010083, EPI_ISL_5010110, EPI_ISL_5010321

see above Puskesmas Galur I Genetics Working Group (Pokja Genetik) Faculty of Medicine, Public Health and Nursing Universitas Gadjah Mada (FK-KMK UGM);

Disease Investigation Center Wates Ministry of Agriculture Indonesia; Department of Microbiology FK-KMK UGM; Laboratorium Diagnostik Yayasan Tahija World Mosquito Program (WMP) Yogyakarta Center for Tropical Medicine FK-KMK UGM; Integrated Research Center FK-KMK UGM; Department of Computer Science and Electronics FMIPA UGM; RSUP Dr. Sardjito

Afiahayati; Alvina A Setiawan; Cita S Amalia; Dwi AA Nugrahaningsih; Dwi Indaryati; Dyah A Puspitarani; Edwin W. Daniwijaya; Eggi Arguni; Endah Supriyati; Gunadi; Hana F Hanifin; Hendra Wibawa; Indarto Sulistiyono; Irene Tania; Khanza a Vujira; Kristy Iskandar; Ludhang P. Rizki; Marcellus; Mohamad S. Hakim; Nungki Anggorowati; Pramesti G Dewi; Siswanto; Susan Simanjaya; Titik Nuryastuti; Tri Wibawa

EPI_ISL_9755988, EPI_ISL_9755989

Puskesmas Gondokusuman Genetics Working Group (Pokja Genetik) Faculty of Medicine, Public Health and Nursing Universitas Gadjah Mada (FK-KMK UGM); Disease Investigation Center Wates Ministry of Agriculture Indonesia; Department of Microbiology FK-KMK UGM; Laboratorium Diagnostik Yayasan Tahija World Mosquito Program (WMP) Yogyakarta Center for Tropical Medicine FK-KMK UGM; Integrated

Research Center FK-KMK UGM; Department of Computer Science and Electronics FMIPA UGM; RSUP Dr. Sardjito

Afiahayati; Dwi AA Nugrahaningsih; Dyah A Puspitarani; Edwin W. Daniwijaya; Eggi Arguni; Endah Supriyati; Fadila D T Utami; Gita C Gabriela; Gunadi; Hendra Wibawa; Ika Trisnawati; Khanza A Vujira; Kristy Iskandar; Lanang Aditama; Laudria S Eryvinka; Ludhang P. Rizki; Mohamad S. Hakim; Nungki Anggorowati; Riat E Khair; Siswanto; Titik Nuryastuti; Tri Wibawa

EPI_ISL_4982801 Puskesmas Grimulyo I Genetics Working Group (Pokja Genetik) Faculty of Medicine, Public Health and Nursing Universitas Gadjah Mada (FK-KMK UGM);

Disease Investigation Center Wates Ministry of Agriculture Indonesia; Department of Microbiology FK-KMK UGM; Laboratorium Diagnostik Yayasan Tahija World Mosquito Program (WMP) Yogyakarta Center for Tropical Medicine FK-KMK UGM; Integrated Research Center FK-KMK UGM; Department of Computer Science and Electronics FMIPA UGM; RSUP Dr. Sardjito

EPI_ISL_5010106 Puskesmas Kalibawang Genetics Working Group (Pokja Genetik) Faculty of Medicine, Public Health and Nursing Universitas Gadjah Mada (FK-KMK UGM);

Disease Investigation Center Wates Ministry of Agriculture Indonesia; Department of Microbiology FK-KMK UGM; Laboratorium Diagnostik Yayasan Tahija World Mosquito Program (WMP) Yogyakarta Center for Tropical Medicine FK-KMK UGM; Integrated Research Center FK-KMK UGM; Department of Computer Science and Electronics FMIPA UGM; RSUP Dr. Sardjito

Afiahayati; Dwi AA Nugrahaningsih; Dwi Indaryati; Edwin W. Daniwijaya; Eggi Arguni; Endah Supriyati; Gunadi; Hana F Hanifin; Hendra Wibawa; Indarto Sulistiyono; Irene Tania; Kristy Iskandar; Ludhang P. Rizki; Marcellus; Mohamad S. Hakim; Nungki Anggorowati; Pramesti G Dewi; Siswanto; Titik Nuryastuti; Tri Wibawa

Afiahayati; Alvina A Setiawan; Cita S Amalia; Dwi AA Nugrahaningsih; Dwi Indaryati; Edwin W. Daniwijaya; Eggi Arguni; Endah Supriyati; Gunadi; Hendra Wibawa; Indarto Sulistiyono; Kristy Iskandar; Ludhang P. Rizki; Marcellus; Mohamad S. Hakim; Nungki Anggorowati; Pramesti G Dewi; Siswanto; Titik Nuryastuti; Tri Wibawa

EPI_ISL_4983057, EPI_ISL_5010024, EPI_ISL_5010104, EPI_ISL_5010355

Puskesmas Kokap II Genetics Working Group (Pokja Genetik) Faculty of Medicine, Public Health and Nursing Universitas Gadjah Mada (FK-KMK UGM); Disease Investigation Center Wates Ministry of Agriculture Indonesia; Department of Microbiology FK-KMK UGM; Laboratorium Diagnostik Yayasan Tahija World Mosquito Program (WMP) Yogyakarta Center for Tropical Medicine FK-KMK UGM; Integrated

Research Center FK-KMK UGM; Department of Computer Science and Electronics FMIPA UGM; RSUP Dr. Sardjito

Afiahayati; Alvina A Setiawan; Cita S Amalia; Dwi AA Nugrahaningsih; Dwi Indaryati; Dyah A Puspitarani; Edwin W. Daniwijaya; Eggi Arguni; Endah Supriyati; Gunadi; Hana F Hanifin; Hendra Wibawa; Indarto Sulistiyono; Irene Tania; Kristy Iskandar; Ludhang P. Rizki; Marcellus; Mohamad S. Hakim; Nungki Anggorowati; Pramesti G Dewi; Siswanto; Susan Simanjaya; Titik Nuryastuti; Tri Wibawa

EPI_ISL_5010073 Puskesmas Lendah I Genetics Working Group (Pokja Genetik) Faculty of Medicine, Public Health and Nursing Universitas Gadjah Mada (FK-KMK UGM);

Disease Investigation Center Wates Ministry of Agriculture Indonesia; Department of Microbiology FK-KMK UGM; Laboratorium Diagnostik Yayasan Tahija World Mosquito Program (WMP) Yogyakarta Center for Tropical Medicine FK-KMK UGM; Integrated Research Center FK-KMK UGM; Department of Computer Science and Electronics FMIPA UGM; RSUP Dr. Sardjito

EPI_ISL_5010492 Puskesmas PLERET Genetics Working Group (Pokja Genetik) Faculty of Medicine, Public Health and Nursing Universitas Gadjah Mada (FK-KMK UGM);

Disease Investigation Center Wates Ministry of Agriculture Indonesia; Department of Microbiology FK-KMK UGM; Laboratorium Diagnostik Yayasan Tahija World Mosquito Program (WMP) Yogyakarta Center for Tropical Medicine FK-KMK UGM; Integrated Research Center FK-KMK UGM; Department of Computer Science and Electronics FMIPA UGM; RSUP Dr. Sardjito

EPI_ISL_4980593 Puskesmas Pengasih I Genetics Working Group (Pokja Genetik) Faculty of Medicine, Public Health and Nursing Universitas Gadjah Mada (FK-KMK UGM);

Disease Investigation Center Wates Ministry of Agriculture Indonesia; Department of Microbiology FK-KMK UGM; Laboratorium Diagnostik Yayasan Tahija World Mosquito Program (WMP) Yogyakarta Center for Tropical Medicine FK-KMK UGM; Integrated Research Center FK-KMK UGM; Department of Computer Science and Electronics FMIPA UGM; RSUP Dr. Sardjito

Afiahayati; Alvina A Setiawan; Cita S Amalia; Dwi AA Nugrahaningsih; Dwi Indaryati; Edwin W. Daniwijaya; Eggi Arguni; Endah Supriyati; Gunadi; Hendra Wibawa; Indarto Sulistiyono; Kristy Iskandar; Ludhang P. Rizki; Marcellus; Mohamad S. Hakim; Nungki Anggorowati; Pramesti G Dewi; Siswanto; Titik Nuryastuti; Tri Wibawa

Afiahayati; Alvina A Setiawan; Dwi AA Nugrahaningsih; Dwi Indaryati; Edwin W. Daniwijaya; Eggi Arguni; Endah Supriyati; Gunadi; Hendra Wibawa; Indarto Sulistiyono; Khanza a Vujira; Kristy Iskandar; Ludhang P. Rizki; Marcellus; Mohamad S. Hakim; Nungki Anggorowati; Pramesti G Dewi; Siswanto; Titik Nuryastuti; Tri Wibawa

Afiahayati; Alvina A Setiawan; Dwi AA Nugrahaningsih; Dwi Indaryati; Edwin W. Daniwijaya; Eggi Arguni; Endah Supriyati; Gunadi; Hendra Wibawa; Indarto Sulistiyono; Khanza a Vujira; Kristy Iskandar; Ludhang P. Rizki; Marcellus; Mohamad S. Hakim; Nungki Anggorowati; Pramesti G Dewi; Siswanto; Titik Nuryastuti; Tri Wibawa

EPI_ISL_4980431, EPI_ISL_4983391

Puskesmas Pengasih II Genetics Working Group (Pokja Genetik) Faculty of Medicine, Public Health and Nursing Universitas Gadjah Mada (FK-KMK UGM); Disease Investigation Center Wates Ministry of Agriculture Indonesia; Department of Microbiology FK-KMK UGM; Laboratorium Diagnostik Yayasan Tahija World Mosquito Program (WMP) Yogyakarta Center for Tropical Medicine FK-KMK UGM; Integrated

Research Center FK-KMK UGM; Department of Computer Science and Electronics FMIPA UGM; RSUP Dr. Sardjito

Afiahayati; Alvina A Setiawan; Cita S Amalia; Dwi AA Nugrahaningsih; Dwi Indaryati; Edwin W. Daniwijaya; Eggi Arguni; Endah Supriyati; Gunadi; Hendra Wibawa; Indarto Sulistiyono; Khanza a Vujira; Kristy Iskandar; Ludhang P. Rizki; Marcellus; Mohamad S. Hakim; Nungki Anggorowati; Pramesti G Dewi; Siswanto; Titik Nuryastuti; Tri Wibawa

EPI_ISL_5010416 Puskesmas Pleret Genetics Working Group (Pokja Genetik) Faculty of Medicine, Public Health and Nursing Universitas Gadjah Mada (FK-KMK UGM);

Disease Investigation Center Wates Ministry of Agriculture Indonesia; Department of Microbiology FK-KMK UGM; Laboratorium Diagnostik Yayasan Tahija World Mosquito Program (WMP) Yogyakarta Center for Tropical Medicine FK-KMK UGM; Integrated Research Center FK-KMK UGM; Department of Computer Science and Electronics FMIPA UGM; RSUP Dr. Sardjito

EPI_ISL_5010063 Puskesmas Samigaluh I Genetics Working Group (Pokja Genetik) Faculty of Medicine, Public Health and Nursing Universitas Gadjah Mada (FK-KMK UGM);

Disease Investigation Center Wates Ministry of Agriculture Indonesia; Department of Microbiology FK-KMK UGM; Laboratorium Diagnostik Yayasan Tahija World Mosquito Program (WMP) Yogyakarta Center for Tropical Medicine FK-KMK UGM; Integrated Research Center FK-KMK UGM; Department of Computer Science and Electronics FMIPA UGM; RSUP Dr. Sardjito

EPI_ISL_4969291 Puskesmas Temon 2 Genetics Working Group (Pokja Genetik) Faculty of Medicine, Public Health and Nursing Universitas Gadjah Mada (FK-KMK UGM);

Disease Investigation Center Wates Ministry of Agriculture Indonesia; Department of Microbiology FK-KMK UGM; Laboratorium Diagnostik Yayasan Tahija World Mosquito Program (WMP) Yogyakarta Center for Tropical Medicine FK-KMK UGM; Integrated Research Center FK-KMK UGM; Department of Computer Science and Electronics FMIPA UGM; RSUP Dr. Sardjito

EPI_ISL_7163388, EPI_ISL_7163917, EPI_ISL_7166591, EPI_ISL_7166628, EPI_ISL_7166791, EPI_ISL_7167384, EPI_ISL_7167528

see above RS Akademik UGM Genetics Working Group (Pokja Genetik) Faculty of Medicine, Public Health and Nursing Universitas Gadjah Mada (FK-KMK UGM);

Disease Investigation Center Wates Ministry of Agriculture Indonesia; Department of Microbiology FK-KMK UGM; Laboratorium Diagnostik Yayasan Tahija World Mosquito Program (WMP) Yogyakarta Center for Tropical Medicine FK-KMK UGM; Integrated Research Center FK-KMK UGM; Department of Computer Science and Electronics FMIPA UGM; RSUP Dr. Sardjito

Afiahayati; Dwi AA Nugrahaningsih; Dwi Indaryati; Dyah A Puspitarani; Edwin W. Daniwijaya; Eggi Arguni; Endah Supriyati; Gunadi; Hendra Wibawa; Indarto Sulistiyono; Kristy Iskandar; Ludhang P. Rizki; Marcellus; Mohamad S. Hakim; Nungki Anggorowati; Pramesti G Dewi; Siswanto; Susan Simanjaya; Titik Nuryastuti; Tri Wibawa

Afiahayati; Dwi AA Nugrahaningsih; Dwi Indaryati; Dyah A Puspitarani; Edwin W. Daniwijaya; Eggi Arguni; Endah Supriyati; Gunadi; Hendra Wibawa; Indarto Sulistiyono; Kristy Iskandar; Ludhang P. Rizki; Marcellus; Mohamad S. Hakim; Nungki Anggorowati; Pramesti G Dewi; Siswanto; Susan Simanjaya; Titik Nuryastuti; Tri Wibawa

Afiahayati; Alvina A Setiawan; Dwi AA Nugrahaningsih; Dwi Indaryati; Edwin W. Daniwijaya; Eggi Arguni; Endah Supriyati; Gunadi; Hendra Wibawa; Indarto Sulistiyono; Khanza a Vujira; Kristy Iskandar; Ludhang P. Rizki; Marcellus; Mohamad S. Hakim; Nungki Anggorowati; Pramesti G Dewi; Siswanto; Titik Nuryastuti; Tri Wibawa

Afiahayati; Dwi AA Nugrahaningsih; Dwi Indaryati; Dyah A Puspitarani; Edwin W. Daniwijaya; Eggi Arguni; Endah Supriyati; Gita C Gabriela; Gunadi; Hendra Wibawa; Indarto Sulistiyono; Khanza A Vujira; Kristy Iskandar; Ludhang P. Rizki; Marcellus; Mohamad S. Hakim; Nungki Anggorowati; Pramesti G Dewi; Siswanto; Susan Simanjaya; Titik Nuryastuti; Tri Wibawa

EPI_ISL_10657734, EPI_ISL_10658766

RS DR. OEN KANDANG SAPI SOLO Genetics Working Group (Pokja Genetik) Faculty of Medicine, Public Health and Nursing Universitas Gadjah Mada (FK-KMK UGM); Disease Investigation Center Wates Ministry of Agriculture Indonesia; Department of Microbiology FK-KMK UGM; Laboratorium Diagnostik Yayasan Tahija World Mosquito Program (WMP) Yogyakarta Center for Tropical Medicine FK-KMK UGM; Integrated

Research Center FK-KMK UGM; Department of Computer Science and Electronics FMIPA UGM; RSUP Dr. Sardjito

Afiahayati; Dwi AA Nugrahaningsih; Dyah A Puspitarani; Edita M Devana; Edwin W. Daniwijaya; Eggi Arguni; Endah Supriyati; Esensi T Geometri; Fadila D T Utami; Gita C Gabriela; Gunadi; Hendra Wibawa; Ika Trisnawati; Khanza A Vujira; Kristy Iskandar; Ludhang P. Rizki; Mohamad S. Hakim; Nungki Anggorowati; Riat E Khair; Siswanto; Titik Nuryastuti; Tri Wibawa; Verrell Christopher

EPI_ISL_11800694 RS Dr. Oen Solo Baru Genetics Working Group (Pokja Genetik) Faculty of Medicine, Public Health and Nursing Universitas Gadjah Mada (FK-KMK UGM);

Disease Investigation Center Wates Ministry of Agriculture Indonesia; BBTKLPP Yogyakarta; Department of Microbiology FK-KMK UGM; Laboratorium Diagnostik Yayasan Tahija World Mosquito Program (WMP) Yogyakarta Center for Tropical Medicine FK-KMK UGM; Integrated Research Center FK-KMK UGM; Department of Computer Science and Electronics FMIPA UGM; RSUP Dr. Sardjito

Afiahayati; Aning; Dwi AA Nugrahaningsih; Dyah A Puspitarani; Edita M Devana; Edwin W. Daniwijaya; Eggi Arguni; Endah Supriyati; Esensi T Geometri; Fadila D T Utami; Fatin; Gita C Gabriela; Gunadi; Hani; Havid; Hendra Wibawa; Ika Trisnawati; Irene; Khanza A Vujira; Kristy Iskandar; Lanang Aditama; Laudria S Eryvinka; Mohamad S. Hakim; Nathania C P Kinasih; Rahmi Nanda; Riat E Khair; Siswanto; Sri Fatmawati; Titik Nuryastuti; Tri Wibawa; Verrell Christopher

EPI_ISL_10639787, EPI_ISL_10657733, EPI_ISL_10658765

RS Dr. Oen Solo Baru Genetics Working Group (Pokja Genetik) Faculty of Medicine, Public Health and Nursing Universitas Gadjah Mada (FK-KMK UGM); Disease Investigation Center Wates Ministry of Agriculture Indonesia; Department of Microbiology FK-KMK UGM; Laboratorium Diagnostik Yayasan Tahija World Mosquito Program (WMP) Yogyakarta Center for Tropical Medicine FK-KMK UGM; Integrated

Research Center FK-KMK UGM; Department of Computer Science and Electronics FMIPA UGM; RSUP Dr. Sardjito

Afiahayati; Dwi AA Nugrahaningsih; Dyah A Puspitarani; Edita M Devana; Edwin W. Daniwijaya; Eggi Arguni; Endah Supriyati; Esensi T Geometri; Fadila D T Utami; Gita C Gabriela; Gunadi; Hendra Wibawa; Ika Trisnawati; Khanza A Vujira; Kristy Iskandar; Ludhang P. Rizki; Mohamad S. Hakim; Nungki Anggorowati; Riat E Khair; Siswanto; Titik Nuryastuti; Tri Wibawa; Verrell Christopher

EPI_ISL_4968087, RS JIH Genetics Working Group (Pokja Genetik) Faculty of Medicine, Public Health and Nursing Universitas Gadjah Mada (FK-KMK UGM); Afiahayati; Alvina A Setiawan; Cita S Amalia; Dwi AA Nugrahaningsih; Dwi Indaryati; Dyah A Puspitarani; Edwin W. Daniwijaya; Eggi Arguni; Endah Supriyati; Gunadi; Hana F Hanifin;

| EPI_ISL_4969010, EPI_ISL_4969082,  EPI_ISL_4969086 |  | Disease Investigation Center Wates Ministry of Agriculture Indonesia; Department of Microbiology FK-KMK UGM; Laboratorium Diagnostik Yayasan Tahija World Mosquito Program (WMP) Yogyakarta Center for Tropical Medicine FK-KMK UGM; Integrated  Research Center FK-KMK UGM; Department of Computer Science and Electronics FMIPA UGM; RSUP Dr. Sardjito | Hendra Wibawa; Indarto Sulistiyono; Irene Tania; Khanza a Vujira; Kristy Iskandar; Ludhang P. Rizki; Marcellus; Mohamad S. Hakim; Nungki Anggorowati; Pramesti G Dewi; Siswanto; Susan Simanjaya; Titik Nuryastuti; Tri Wibawa |
| --- | --- | --- | --- |
| EPI_ISL_10658764 | RS KASIH IBU | Genetics Working Group (Pokja Genetik) Faculty of Medicine, Public Health and Nursing Universitas Gadjah Mada (FK-KMK UGM); Disease Investigation Center Wates Ministry of Agriculture Indonesia; Department of Microbiology FK-KMK UGM; Laboratorium Diagnostik Yayasan Tahija World Mosquito Program (WMP) Yogyakarta Center for Tropical Medicine FK-KMK UGM; Integrated Research Center FK-KMK UGM; Department of Computer Science and Electronics FMIPA UGM; RSUP Dr. Sardjito | Afiahayati; Dwi AA Nugrahaningsih; Dyah A Puspitarani; Edita M Devana; Edwin W. Daniwijaya; Eggi Arguni; Endah Supriyati; Esensi T Geometri; Fadila D T Utami; Gita C Gabriela; Gunadi; Hendra Wibawa; Ika Trisnawati; Khanza A Vujira; Kristy Iskandar; Ludhang P. Rizki; Mohamad S. Hakim; Nungki Anggorowati; Riat E Khair; Siswanto; Titik Nuryastuti; Tri Wibawa; Verrell Christopher |
| EPI_ISL_10640044 | RS KASIH IBU SURAKARTA | Genetics Working Group (Pokja Genetik) Faculty of Medicine, Public Health and Nursing Universitas Gadjah Mada (FK-KMK UGM); Disease Investigation Center Wates Ministry of Agriculture Indonesia; Department of Microbiology FK-KMK UGM; Laboratorium Diagnostik Yayasan Tahija World Mosquito Program (WMP) Yogyakarta Center for Tropical Medicine FK-KMK UGM; Integrated Research Center FK-KMK UGM; Department of Computer Science and Electronics FMIPA UGM; RSUP Dr. Sardjito | Afiahayati; Dwi AA Nugrahaningsih; Dyah A Puspitarani; Edita M Devana; Edwin W. Daniwijaya; Eggi Arguni; Endah Supriyati; Esensi T Geometri; Fadila D T Utami; Gita C Gabriela; Gunadi; Hendra Wibawa; Ika Trisnawati; Khanza A Vujira; Kristy Iskandar; Ludhang P. Rizki; Mohamad S. Hakim; Nungki Anggorowati; Riat E Khair; Siswanto; Titik Nuryastuti; Tri Wibawa; Verrell Christopher |
| EPI_ISL_9755959 | RS PKU Gamping | Genetics Working Group (Pokja Genetik) Faculty of Medicine, Public Health and Nursing Universitas Gadjah Mada (FK-KMK UGM); Disease Investigation Center Wates Ministry of Agriculture Indonesia; Department of Microbiology FK-KMK UGM; Laboratorium Diagnostik Yayasan Tahija World Mosquito Program (WMP) Yogyakarta Center for Tropical Medicine FK-KMK UGM; Integrated Research Center FK-KMK UGM; Department of Computer Science and Electronics FMIPA UGM; RSUP Dr. Sardjito | Afiahayati; Dwi AA Nugrahaningsih; Dyah A Puspitarani; Edwin W. Daniwijaya; Eggi Arguni; Endah Supriyati; Fadila D T Utami; Gita C Gabriela; Gunadi; Hendra Wibawa; Ika Trisnawati; Khanza A Vujira; Kristy Iskandar; Lanang Aditama; Laudria S Eryvinka; Ludhang P. Rizki; Mohamad S. Hakim; Nungki Anggorowati; Riat E Khair; Siswanto; Titik Nuryastuti; Tri Wibawa |
| EPI_ISL_9713702 | RS PKU Kota Yogyakarta | Genetics Working Group (Pokja Genetik) Faculty of Medicine, Public Health and Nursing Universitas Gadjah Mada (FK-KMK UGM); Disease Investigation Center Wates Ministry of Agriculture Indonesia; Department of Microbiology FK-KMK UGM; Laboratorium Diagnostik Yayasan Tahija World Mosquito Program (WMP) Yogyakarta Center for Tropical Medicine FK-KMK UGM; Integrated Research Center FK-KMK UGM; Department of Computer Science and Electronics FMIPA UGM; RSUP Dr. Sardjito | Afiahayati; Dwi AA Nugrahaningsih; Dyah A Puspitarani; Edwin W. Daniwijaya; Eggi Arguni; Endah Supriyati; Fadila D T Utami; Gita C Gabriela; Gunadi; Hendra Wibawa; Ika Trisnawati; Khanza A Vujira; Kristy Iskandar; Lanang Aditama; Laudria S Eryvinka; Ludhang P. Rizki; Mohamad S. Hakim; Nungki Anggorowati; Riat E Khair; Siswanto; Titik Nuryastuti; Tri Wibawa |
| EPI_ISL_9713704 | RS PKU Muh. Gamping | Genetics Working Group (Pokja Genetik) Faculty of Medicine, Public Health and Nursing Universitas Gadjah Mada (FK-KMK UGM); Disease Investigation Center Wates Ministry of Agriculture Indonesia; Department of Microbiology FK-KMK UGM; Laboratorium Diagnostik Yayasan Tahija World Mosquito Program (WMP) Yogyakarta Center for Tropical Medicine FK-KMK UGM; Integrated Research Center FK-KMK UGM; Department of Computer Science and Electronics FMIPA UGM; RSUP Dr. Sardjito | Afiahayati; Dwi AA Nugrahaningsih; Dyah A Puspitarani; Edwin W. Daniwijaya; Eggi Arguni; Endah Supriyati; Fadila D T Utami; Gita C Gabriela; Gunadi; Hendra Wibawa; Ika Trisnawati; Khanza A Vujira; Kristy Iskandar; Lanang Aditama; Laudria S Eryvinka; Ludhang P. Rizki; Mohamad S. Hakim; Nungki Anggorowati; Riat E Khair; Siswanto; Titik Nuryastuti; Tri Wibawa |
| EPI_ISL_9487456 | RS PKU Muhammadiyah Bantul | Genetics Working Group (Pokja Genetik) Faculty of Medicine, Public Health and Nursing Universitas Gadjah Mada (FK-KMK UGM); Disease Investigation Center Wates Ministry of Agriculture Indonesia; Department of Microbiology FK-KMK UGM; Laboratorium Diagnostik Yayasan Tahija World Mosquito Program (WMP) Yogyakarta Center for Tropical Medicine FK-KMK UGM; Integrated Research Center FK-KMK UGM; Department of Computer Science and Electronics FMIPA UGM; RSUP Dr. Sardjito | Afiahayati; Dwi AA Nugrahaningsih; Dyah A Puspitarani; Edwin W. Daniwijaya; Eggi Arguni; Endah Supriyati; Fadila D T Utami; Gita C Gabriela; Gunadi; Hendra Wibawa; Ika Trisnawati; Khanza A Vujira; Kristy Iskandar; Lanang Aditama; Laudria S Eryvinka; Ludhang P. Rizki; Mohamad S. Hakim; Nungki Anggorowati; Riat E Khair; Siswanto; Titik Nuryastuti; Tri Wibawa |
| EPI_ISL_9433335, EPI_ISL_9434455 | RS Panti Rapih | Genetics Working Group (Pokja Genetik) Faculty of Medicine, Public Health and Nursing Universitas Gadjah Mada (FK-KMK UGM); Disease Investigation Center Wates Ministry of Agriculture Indonesia; Department of Microbiology FK-KMK UGM; Laboratorium Diagnostik Yayasan Tahija World Mosquito Program (WMP) Yogyakarta Center for Tropical Medicine FK-KMK UGM; Integrated Research Center FK-KMK UGM; Department of Computer Science and Electronics FMIPA UGM; RSUP Dr. Sardjito | Afiahayati; Dwi AA Nugrahaningsih; Dwi Indaryati; Dyah A Puspitarani; Edwin W. Daniwijaya; Eggi Arguni; Endah Supriyati; Gunadi; Hendra Wibawa; Indarto Sulistiyono; Khanza A Vujira; Kristy Iskandar; Ludhang P. Rizki; Mohamad S. Hakim; Nungki Anggorowati; Pramesti G Dewi; Siswanto; Titik Nuryastuti; Tri Wibawa |
| EPI_ISL_5010527 | RS Rajawali Citra | Genetics Working Group (Pokja Genetik) Faculty of Medicine, Public Health and Nursing Universitas Gadjah Mada (FK-KMK UGM); Disease Investigation Center Wates Ministry of Agriculture Indonesia; Department of Microbiology FK-KMK UGM; Laboratorium Diagnostik Yayasan Tahija World Mosquito Program (WMP) Yogyakarta Center for Tropical Medicine FK-KMK UGM; Integrated Research Center FK-KMK UGM; Department of Computer Science and Electronics FMIPA UGM; RSUP Dr. Sardjito | Afiahayati; Alvina A Setiawan; Cita S Amalia; Dwi AA Nugrahaningsih; Dwi Indaryati; Edwin W. Daniwijaya; Eggi Arguni; Endah Supriyati; Gunadi; Hendra Wibawa; Indarto Sulistiyono; Kristy Iskandar; Ludhang P. Rizki; Marcellus; Mohamad S. Hakim; Nungki Anggorowati; Pramesti G Dewi; Siswanto; Titik Nuryastuti; Tri Wibawa |
| EPI_ISL_10969463, EPI_ISL_10969464 | RS. Pertamina Cilacap | Genetics Working Group (Pokja Genetik) Faculty of Medicine, Public Health and Nursing Universitas Gadjah Mada (FK-KMK UGM); Balai Besar Teknik Kesehatan Lingkungan Dan Pengendalian Penyakit (BBTKLPP) Yogyakarta; Department of Microbiology FK-KMK UGM; Laboratorium Diagnostik Yayasan Tahija World Mosquito Program (WMP) Yogyakarta Center for Tropical Medicine FK-KMK UGM; Integrated Research Center FK-KMK UGM; Department of Computer Science and Electronics FMIPA UGM; RSUP Dr. Sardjito | Afiahayati; Aning; Dwi AA Nugrahaningsih; Dyah A Puspitarani; Edita M Devana; Edwin W. Daniwijaya; Eggi Arguni; Endah Supriyati; Esensi T Geometri; Fadila D T Utami; Fatin; Gita C Gabriela; Gunadi; Hani; Havid; Hendra Wibawa; Ika Trisnawati; Irene; Khanza A Vujira; Kristy Iskandar; Lanang Aditama; Laudria S Eryvinka; Mohamad S. Hakim; Nathania C P Kinasih; Rahmi Nanda; Riat E Khair; Siswanto; Sri Fatmawati; Titik Nuryastuti; Tri Wibawa; Verrell Christopher |
| EPI_ISL_10969465, EPI_ISL_10969466 | RSA UGM | Genetics Working Group (Pokja Genetik) Faculty of Medicine, Public Health and Nursing Universitas Gadjah Mada (FK-KMK UGM); Balai Besar Teknik Kesehatan Lingkungan Dan Pengendalian Penyakit (BBTKLPP) Yogyakarta; Department of Microbiology FK-KMK UGM; Laboratorium Diagnostik Yayasan Tahija World Mosquito Program (WMP) Yogyakarta Center for Tropical Medicine FK-KMK UGM; Integrated Research Center FK-KMK UGM; Department of Computer Science and Electronics FMIPA UGM; RSUP Dr. Sardjito | Afiahayati; Aning; Dwi AA Nugrahaningsih; Dyah A Puspitarani; Edita M Devana; Edwin W. Daniwijaya; Eggi Arguni; Endah Supriyati; Esensi T Geometri; Fadila D T Utami; Fatin; Gita C Gabriela; Gunadi; Hani; Havid; Hendra Wibawa; Ika Trisnawati; Irene; Khanza A Vujira; Kristy Iskandar; Lanang Aditama; Laudria S Eryvinka; Mohamad S. Hakim; Nathania C P Kinasih; Rahmi Nanda; Riat E Khair; Siswanto; Sri Fatmawati; Titik Nuryastuti; Tri Wibawa; Verrell Christopher |

EPI_ISL_7162211, EPI_ISL_7162387, EPI_ISL_7162539, EPI_ISL_7162961, EPI_ISL_7163289, EPI_ISL_7163308, EPI_ISL_7163385, EPI_ISL_9434592, EPI_ISL_9434818, EPI_ISL_9487464, EPI_ISL_12241066

see above RSA UGM Genetics Working Group (Pokja Genetik) Faculty of Medicine, Public Health and Nursing Universitas Gadjah Mada (FK-KMK UGM);

Disease Investigation Center Wates Ministry of Agriculture Indonesia; Department of Microbiology FK-KMK UGM; Laboratorium Diagnostik Yayasan Tahija World Mosquito Program (WMP) Yogyakarta Center for Tropical Medicine FK-KMK UGM; Integrated Research Center FK-KMK UGM; Department of Computer Science and Electronics FMIPA UGM; RSUP Dr. Sardjito

EPI_ISL_4969084 RSI NU Demak Genetics Working Group (Pokja Genetik) Faculty of Medicine, Public Health and Nursing Universitas Gadjah Mada (FK-KMK UGM);

Disease Investigation Center Wates Ministry of Agriculture Indonesia; Department of Microbiology FK-KMK UGM; Laboratorium Diagnostik Yayasan Tahija World Mosquito Program (WMP) Yogyakarta Center for Tropical Medicine FK-KMK UGM; Integrated Research Center FK-KMK UGM; Department of Computer Science and Electronics FMIPA UGM; RSUP Dr. Sardjito

EPI_ISL_7166965 RSKIA Sadewa Genetics Working Group (Pokja Genetik) Faculty of Medicine, Public Health and Nursing Universitas Gadjah Mada (FK-KMK UGM);

Disease Investigation Center Wates Ministry of Agriculture Indonesia; Department of Microbiology FK-KMK UGM; Laboratorium Diagnostik Yayasan Tahija World Mosquito Program (WMP) Yogyakarta Center for Tropical Medicine FK-KMK UGM; Integrated Research Center FK-KMK UGM; Department of Computer Science and Electronics FMIPA UGM; RSUP Dr. Sardjito

EPI_ISL_9487465 RSPAU dr. S.Hardjolukito Genetics Working Group (Pokja Genetik) Faculty of Medicine, Public Health and Nursing Universitas Gadjah Mada (FK-KMK UGM);

Disease Investigation Center Wates Ministry of Agriculture Indonesia; Department of Microbiology FK-KMK UGM; Laboratorium Diagnostik Yayasan Tahija World Mosquito Program (WMP) Yogyakarta Center for Tropical Medicine FK-KMK UGM; Integrated Research Center FK-KMK UGM; Department of Computer Science and Electronics FMIPA UGM; RSUP Dr. Sardjito

Afiahayati; Dwi AA Nugrahaningsih; Dwi Indaryati; Dyah A Puspitarani; Edwin W. Daniwijaya; Eggi Arguni; Endah Supriyati; Fadila D T Utami; Gita C Gabriela; Gunadi; Hendra Wibawa; Ika Trisnawati; Indarto Sulistiyono; Khanza A Vujira; Kristy Iskandar; Lanang Aditama; Laudria S Eryvinka; Ludhang P. Rizki; Marcellus; Mohamad S. Hakim; Nungki Anggorowati; Pramesti G Dewi; Riat E Khair; Siswanto; Susan Simanjaya; Titik Nuryastuti; Tri Wibawa

Afiahayati; Alvina A Setiawan; Dwi AA Nugrahaningsih; Dwi Indaryati; Edwin W. Daniwijaya; Eggi Arguni; Endah Supriyati; Gunadi; Hendra Wibawa; Indarto Sulistiyono; Khanza a Vujira; Kristy Iskandar; Ludhang P. Rizki; Marcellus; Mohamad S. Hakim; Nungki Anggorowati; Pramesti G Dewi; Siswanto; Titik Nuryastuti; Tri Wibawa

Afiahayati; Dwi AA Nugrahaningsih; Dwi Indaryati; Dyah A Puspitarani; Edwin W. Daniwijaya; Eggi Arguni; Endah Supriyati; Gita C Gabriela; Gunadi; Hendra Wibawa; Indarto Sulistiyono; Khanza A Vujira; Kristy Iskandar; Ludhang P. Rizki; Marcellus; Mohamad S. Hakim; Nungki Anggorowati; Pramesti G Dewi; Siswanto; Titik Nuryastuti; Tri Wibawa

Afiahayati; Dwi AA Nugrahaningsih; Dyah A Puspitarani; Edwin W. Daniwijaya; Eggi Arguni; Endah Supriyati; Fadila D T Utami; Gita C Gabriela; Gunadi; Hendra Wibawa; Ika Trisnawati; Khanza A Vujira; Kristy Iskandar; Lanang Aditama; Laudria S Eryvinka; Ludhang P. Rizki; Mohamad S. Hakim; Nungki Anggorowati; Riat E Khair; Siswanto; Titik Nuryastuti; Tri Wibawa

EPI_ISL_9702414, EPI_ISL_9702417, EPI_ISL_9713700, EPI_ISL_9713701

RSPAU dr.S.Hardjolukito Genetics Working Group (Pokja Genetik) Faculty of Medicine, Public Health and Nursing Universitas Gadjah Mada (FK-KMK UGM); Disease Investigation Center Wates Ministry of Agriculture Indonesia; Department of Microbiology FK-KMK UGM; Laboratorium Diagnostik Yayasan Tahija World Mosquito Program (WMP) Yogyakarta Center for Tropical Medicine FK-KMK UGM; Integrated

Research Center FK-KMK UGM; Department of Computer Science and Electronics FMIPA UGM; RSUP Dr. Sardjito

Afiahayati; Dwi AA Nugrahaningsih; Dyah A Puspitarani; Edwin W. Daniwijaya; Eggi Arguni; Endah Supriyati; Fadila D T Utami; Gita C Gabriela; Gunadi; Hendra Wibawa; Ika Trisnawati; Khanza A Vujira; Kristy Iskandar; Lanang Aditama; Laudria S Eryvinka; Ludhang P. Rizki; Mohamad S. Hakim; Nungki Anggorowati; Riat E Khair; Siswanto; Titik Nuryastuti; Tri Wibawa

EPI_ISL_7160962 RSST Klaten Genetics Working Group (Pokja Genetik) Faculty of Medicine, Public Health and Nursing Universitas Gadjah Mada (FK-KMK UGM);

Disease Investigation Center Wates Ministry of Agriculture Indonesia; Department of Microbiology FK-KMK UGM; Laboratorium Diagnostik Yayasan Tahija World Mosquito Program (WMP) Yogyakarta Center for Tropical Medicine FK-KMK UGM; Integrated Research Center FK-KMK UGM; Department of Computer Science and Electronics FMIPA UGM; RSUP Dr. Sardjito

Afiahayati; Dwi AA Nugrahaningsih; Dwi Indaryati; Dyah A Puspitarani; Edwin W. Daniwijaya; Eggi Arguni; Endah Supriyati; Gita C Gabriela; Gunadi; Hendra Wibawa; Indarto Sulistiyono; Khanza a Vujira; Kristy Iskandar; Ludhang P. Rizki; Marcellus; Mohamad S. Hakim; Nungki Anggorowati; Pramesti G Dewi; Siswanto; Titik Nuryastuti; Tri Wibawa

EPI_ISL_10969451, EPI_ISL_10969452, EPI_ISL_10969453, EPI_ISL_10969454, EPI_ISL_10969455

EPI_ISL_10969460, EPI_ISL_10969461, EPI_ISL_10969462

EPI_ISL_10640043, EPI_ISL_12240910

RSU AISYIYAH Genetics Working Group (Pokja Genetik) Faculty of Medicine, Public Health and Nursing Universitas Gadjah Mada (FK-KMK UGM); Balai Besar Teknik Kesehatan Lingkungan Dan Pengendalian Penyakit (BBTKLPP) Yogyakarta; Department of Microbiology FK-KMK

UGM; Laboratorium Diagnostik Yayasan Tahija World Mosquito Program (WMP) Yogyakarta Center for Tropical Medicine FK-KMK UGM; Integrated Research Center FK-KMK UGM; Department of Computer Science and Electronics FMIPA UGM; RSUP Dr. Sardjito

RSUD Wonogiri Genetics Working Group (Pokja Genetik) Faculty of Medicine, Public Health and Nursing Universitas Gadjah Mada (FK-KMK UGM); Balai Besar Teknik Kesehatan Lingkungan Dan Pengendalian Penyakit (BBTKLPP) Yogyakarta; Department of Microbiology FK-KMK

UGM; Laboratorium Diagnostik Yayasan Tahija World Mosquito Program (WMP) Yogyakarta Center for Tropical Medicine FK-KMK UGM; Integrated Research Center FK-KMK UGM; Department of Computer Science and Electronics FMIPA UGM; RSUP Dr. Sardjito

RSUD Ibu Fatmawati Soekarno Genetics Working Group (Pokja Genetik) Faculty of Medicine, Public Health and Nursing Universitas Gadjah Mada (FK-KMK UGM); Disease Investigation Center Wates Ministry of Agriculture Indonesia; Department of Microbiology FK-KMK UGM; Laboratorium Diagnostik Yayasan Tahija World Mosquito Program (WMP) Yogyakarta Center for Tropical Medicine FK-KMK UGM; Integrated

Research Center FK-KMK UGM; Department of Computer Science and Electronics FMIPA UGM; RSUP Dr. Sardjito

Afiahayati; Aning; Dwi AA Nugrahaningsih; Dyah A Puspitarani; Edita M Devana; Edwin W. Daniwijaya; Eggi Arguni; Endah Supriyati; Esensi T Geometri; Fadila D T Utami; Fatin; Gita C Gabriela; Gunadi; Hani; Havid; Hendra Wibawa; Ika Trisnawati; Irene; Khanza A Vujira; Kristy Iskandar; Lanang Aditama; Laudria S Eryvinka; Mohamad S. Hakim; Nathania C P Kinasih; Rahmi Nanda; Riat E Khair; Siswanto; Sri Fatmawati; Titik Nuryastuti; Tri Wibawa; Verrell Christopher

Afiahayati; Aning; Dwi AA Nugrahaningsih; Dyah A Puspitarani; Edita M Devana; Edwin W. Daniwijaya; Eggi Arguni; Endah Supriyati; Esensi T Geometri; Fadila D T Utami; Fatin; Gita C Gabriela; Gunadi; Hani; Havid; Hendra Wibawa; Ika Trisnawati; Irene; Khanza A Vujira; Kristy Iskandar; Lanang Aditama; Laudria S Eryvinka; Mohamad S. Hakim; Nathania C P Kinasih; Rahmi Nanda; Riat E Khair; Siswanto; Sri Fatmawati; Titik Nuryastuti; Tri Wibawa; Verrell Christopher

Afiahayati; Dwi AA Nugrahaningsih; Dyah A Puspitarani; Edita M Devana; Edwin W. Daniwijaya; Eggi Arguni; Endah Supriyati; Esensi T Geometri; Fadila D T Utami; Gita C Gabriela; Gunadi; Hendra Wibawa; Ika Trisnawati; Khanza A Vujira; Kristy Iskandar; Ludhang P. Rizki; Mohamad S. Hakim; Nungki Anggorowati; Riat E Khair; Siswanto; Titik Nuryastuti; Tri Wibawa; Verrell Christopher

EPI_ISL_7160960, EPI_ISL_7160961, EPI_ISL_7160982, EPI_ISL_7160983, EPI_ISL_7161078, EPI_ISL_7161365, EPI_ISL_7161601, EPI_ISL_7161977

see above RSUD K.M.R.T Wongsonegoro Genetics Working Group (Pokja Genetik) Faculty of Medicine, Public Health and Nursing Universitas Gadjah Mada (FK-KMK UGM);

Disease Investigation Center Wates Ministry of Agriculture Indonesia; Department of Microbiology FK-KMK UGM; Laboratorium Diagnostik Yayasan Tahija World Mosquito Program (WMP) Yogyakarta Center for Tropical Medicine FK-KMK UGM; Integrated Research Center FK-KMK UGM; Department of Computer Science and Electronics FMIPA UGM; RSUP Dr. Sardjito

. Marcellus; Afiahayati; Bambang Sigit Riyanto; Dwi AA Nugrahaningsih; Dwi Indaryati; Dyah A Puspitarani; Edwin W. Daniwijaya; Eggi Arguni; Eko Budiono; Endah Supriyati; Gita C Gabriela; Gita C Gabriela; Gunadi; Hendra Wibawa; Heni Retnowulan; Ika Trisnawati; Indarto Sulistiyono; Ira Puspitawati; Khanza A Vujira; Khanza a Vujira; Kristy Iskandar; Ludhang P. Rizki; Marcellus; Mohamad S. Hakim; Munawar Gani; Nungki Anggorowati; Nur Imma Fatimah Harahap; Nur Rahmi Ananda; Osman Sianipar; Pramesti G Dewi; Riat El Khair; Satria Maulana; Siswanto; Sumardi; Susan Simanjaya; Titik Nuryastuti; Tri Wibawa; Umi Solekhah Intansari; Yunika Puspadewi; Elizabeth Henny Herningtiyas

EPI_ISL_10639788, EPI_ISL_10639789, EPI_ISL_10657581, EPI_ISL_10658763, EPI_ISL_10658769

EPI_ISL_4969091, EPI_ISL_4969096, EPI_ISL_4969115, EPI_ISL_4969119

EPI_ISL_10969449, EPI_ISL_10969450, EPI_ISL_10969456, EPI_ISL_10969457,

RSUD KRT. SETJONEGORO Genetics Working Group (Pokja Genetik) Faculty of Medicine, Public Health and Nursing Universitas Gadjah Mada (FK-KMK UGM); Disease Investigation Center Wates Ministry of Agriculture Indonesia; Department of Microbiology FK-KMK UGM; Laboratorium Diagnostik Yayasan Tahija World Mosquito Program (WMP) Yogyakarta Center for Tropical Medicine FK-KMK UGM; Integrated

Research Center FK-KMK UGM; Department of Computer Science and Electronics FMIPA UGM; RSUP Dr. Sardjito

RSUD KRT. Setjonegoro Wonosobo Genetics Working Group (Pokja Genetik) Faculty of Medicine, Public Health and Nursing Universitas Gadjah Mada (FK-KMK UGM); Disease Investigation Center Wates Ministry of Agriculture Indonesia; Department of Microbiology FK-KMK UGM; Laboratorium Diagnostik Yayasan Tahija World Mosquito Program (WMP) Yogyakarta Center for Tropical Medicine FK-KMK UGM; Integrated

Research Center FK-KMK UGM; Department of Computer Science and Electronics FMIPA UGM; RSUP Dr. Sardjito

RSUD MUNTILAN Genetics Working Group (Pokja Genetik) Faculty of Medicine, Public Health and Nursing Universitas Gadjah Mada (FK-KMK UGM); Balai Besar Teknik Kesehatan Lingkungan Dan Pengendalian Penyakit (BBTKLPP) Yogyakarta; Department of Microbiology FK-KMK

UGM; Laboratorium Diagnostik Yayasan Tahija World Mosquito Program (WMP) Yogyakarta Center for Tropical Medicine FK-KMK UGM; Integrated Research Center FK-KMK UGM; Department of Computer Science and Electronics FMIPA UGM; RSUP Dr. Sardjito

Afiahayati; Dwi AA Nugrahaningsih; Dyah A Puspitarani; Edita M Devana; Edwin W. Daniwijaya; Eggi Arguni; Endah Supriyati; Esensi T Geometri; Fadila D T Utami; Gita C Gabriela; Gunadi; Hendra Wibawa; Ika Trisnawati; Khanza A Vujira; Kristy Iskandar; Ludhang P. Rizki; Mohamad S. Hakim; Nungki Anggorowati; Riat E Khair; Siswanto; Titik Nuryastuti; Tri Wibawa; Verrell Christopher

. Marcellus; Afiahayati; Alvina A Setiawan; Bambang Sigit Riyanto; Dwi AA Nugrahaningsih; Dwi Indaryati; Dyah A Puspitarani; Edwin W. Daniwijaya; Eggi Arguni; Eko Budiono; Endah Supriyati; Gunadi; Hana F Hanifin; Hendra Wibawa; Heni Retnowulan; Ika Trisnawati; Indarto Sulistiyono; Ira Puspitawati; Irene Tania; Khanza a Vujira; Kristy Iskandar; Ludhang P. Rizki; Marcellus; Mohamad S. Hakim; Munawar Gani; Nungki Anggorowati; Nur Imma Fatimah Harahap; Nur Rahmi Ananda; Osman Sianipar; Pramesti G Dewi; Riat El Khair; Satria Maulana; Siswanto; Sumardi; Susan Simanjaya; Titik Nuryastuti; Tri Wibawa; Umi Solekhah Intansari; Yunika Puspadewi; Elizabeth Henny Herningtiyas

Afiahayati; Aning; Dwi AA Nugrahaningsih; Dyah A Puspitarani; Edita M Devana; Edwin W. Daniwijaya; Eggi Arguni; Endah Supriyati; Esensi T Geometri; Fadila D T Utami; Fatin; Gita C Gabriela; Gunadi; Hani; Havid; Hendra Wibawa; Ika Trisnawati; Irene; Khanza A Vujira; Kristy Iskandar; Lanang Aditama; Laudria S Eryvinka; Mohamad S. Hakim; Nathania C P Kinasih; Rahmi Nanda; Riat E Khair; Siswanto; Sri Fatmawati; Titik Nuryastuti; Tri Wibawa; Verrell Christopher

EPI_ISL_10969458, EPI_ISL_10969459

EPI_ISL_4978455, EPI_ISL_4980931, EPI_ISL_4981198, EPI_ISL_4981563, EPI_ISL_5010016, EPI_ISL_5010036, EPI_ISL_5010038, EPI_ISL_5010046, EPI_ISL_5010054, EPI_ISL_5010412

see above RSUD Nyi Ageng Serang Genetics Working Group (Pokja Genetik) Faculty of Medicine, Public Health and Nursing Universitas Gadjah Mada (FK-KMK UGM);

Disease Investigation Center Wates Ministry of Agriculture Indonesia; Department of Microbiology FK-KMK UGM; Laboratorium Diagnostik Yayasan Tahija World Mosquito Program (WMP) Yogyakarta Center for Tropical Medicine FK-KMK UGM; Integrated Research Center FK-KMK UGM; Department of Computer Science and Electronics FMIPA UGM; RSUP Dr. Sardjito

. Marcellus; Afiahayati; Alvina A Setiawan; Bambang Sigit Riyanto; Cita S Amalia; Dwi AA Nugrahaningsih; Dwi Indaryati; Dyah A Puspitarani; Edwin W. Daniwijaya; Eggi Arguni; Eko Budiono; Endah Supriyati; Gunadi; Hana F Hanifin; Hendra Wibawa; Heni Retnowulan; I Putu Aditio A; Ika Trisnawati; Indarto Sulistiyono; Ira Puspitawati; Irene Tania; Khanza a Vujira; Kristy Iskandar; Ludhang P. Rizki; Marcellus; Mohamad S. Hakim; Munawar Gani; Nungki Anggorowati; Nur Imma Fatimah Harahap; Nur Rahmi Ananda; Osman Sianipar; Pramesti G Dewi; Riat El Khair; Satria Maulana; Siswanto; Sumardi; Susan Simanjaya; Titik Nuryastuti; Tri Wibawa; Umi Solekhah Intansari; Yunika Puspadewi; Elizabeth Henny Herningtiyas

EPI_ISL_4969177, EPI_ISL_4969179

EPI_ISL_7153225, EPI_ISL_7153586

RSUD Sunan Kalijaga Demak Genetics Working Group (Pokja Genetik) Faculty of Medicine, Public Health and Nursing Universitas Gadjah Mada (FK-KMK UGM); Disease Investigation Center Wates Ministry of Agriculture Indonesia; Department of Microbiology FK-KMK UGM; Laboratorium Diagnostik Yayasan Tahija World Mosquito Program (WMP) Yogyakarta Center for Tropical Medicine FK-KMK UGM; Integrated

Research Center FK-KMK UGM; Department of Computer Science and Electronics FMIPA UGM; RSUP Dr. Sardjito

RSUD dr. Loekmono Hadi Genetics Working Group (Pokja Genetik) Faculty of Medicine, Public Health and Nursing Universitas Gadjah Mada (FK-KMK UGM); Disease Investigation Center Wates Ministry of Agriculture Indonesia; Department of Microbiology FK-KMK UGM; Laboratorium Diagnostik Yayasan Tahija World Mosquito Program (WMP) Yogyakarta Center for Tropical Medicine FK-KMK UGM; Integrated

Research Center FK-KMK UGM; Department of Computer Science and Electronics FMIPA UGM; RSUP Dr. Sardjito

Afiahayati; Alvina A Setiawan; Dwi AA Nugrahaningsih; Dwi Indaryati; Dyah A Puspitarani; Edwin W. Daniwijaya; Eggi Arguni; Endah Supriyati; Gunadi; Hendra Wibawa; Indarto Sulistiyono; Khanza a Vujira; Kristy Iskandar; Ludhang P. Rizki; Marcellus; Mohamad S. Hakim; Nungki Anggorowati; Pramesti G Dewi; Siswanto; Susan Simanjaya; Titik Nuryastuti; Tri Wibawa

Afiahayati; Dwi AA Nugrahaningsih; Dwi Indaryati; Dyah A Puspitarani; Edwin W. Daniwijaya; Eggi Arguni; Endah Supriyati; Gita C Gabriela; Gunadi; Hendra Wibawa; Indarto Sulistiyono; Khanza A Vujira; Kristy Iskandar; Ludhang P. Rizki; Marcellus; Mohamad S. Hakim; Nungki Anggorowati; Pramesti G Dewi; Siswanto; Susan Simanjaya; Titik Nuryastuti; Tri Wibawa

EPI_ISL_4969112 RSUP Dr Sardjito Genetics Working Group (Pokja Genetik) Faculty of Medicine, Public Health and Nursing Universitas Gadjah Mada (FK-KMK UGM);

Disease Investigation Center Wates Ministry of Agriculture Indonesia; Department of Microbiology FK-KMK UGM; Laboratorium Diagnostik Yayasan Tahija World Mosquito Program (WMP) Yogyakarta Center for Tropical Medicine FK-KMK UGM; Integrated Research Center FK-KMK UGM; Department of Computer Science and Electronics FMIPA UGM; RSUP Dr. Sardjito

Afiahayati; Dwi AA Nugrahaningsih; Dwi Indaryati; Edwin W. Daniwijaya; Eggi Arguni; Endah Supriyati; Gunadi; Hana F Hanifin; Hendra Wibawa; Indarto Sulistiyono; Irene Tania; Kristy Iskandar; Ludhang P. Rizki; Marcellus; Mohamad S. Hakim; Nungki Anggorowati; Pramesti G Dewi; Siswanto; Titik Nuryastuti; Tri Wibawa

EPI_ISL_10626964, EPI_ISL_10626972, EPI_ISL_10626973, EPI_ISL_10626974, EPI_ISL_10626975, EPI_ISL_10626976, EPI_ISL_10626977, EPI_ISL_10626978, EPI_ISL_10626979, EPI_ISL_10626980, EPI_ISL_10626981, EPI_ISL_10627013, EPI_ISL_10627014

see above RSUP Dr. Sardjito Genetics Working Group (Pokja Genetik) Faculty of Medicine, Public Health and Nursing Universitas Gadjah Mada (FK-KMK UGM);

Disease Investigation Center Wates Ministry of Agriculture Indonesia; Department of Microbiology FK-KMK UGM; Laboratorium Diagnostik Yayasan Tahija World Mosquito Program (WMP) Yogyakarta Center for Tropical Medicine FK-KMK UGM; Integrated Research Center FK-KMK UGM; Department of Computer Science and Electronics FMIPA UGM; RSUP Dr. Sardjito

Afiahayati; Dwi AA Nugrahaningsih; Dyah A Puspitarani; Edita M Devana; Edwin W. Daniwijaya; Eggi Arguni; Endah Supriyati; Esensi T Geometri; Fadila D T Utami; Gita C Gabriela; Gunadi; Hendra Wibawa; Ika Trisnawati; Khanza A Vujira; Kristy Iskandar; Ludhang P. Rizki; Mohamad S. Hakim; Nungki Anggorowati; Riat E Khair; Siswanto; Titik Nuryastuti; Tri Wibawa; Verrell Christopher

EPI_ISL_10969467, EPI_ISL_10969468, EPI_ISL_10969469, EPI_ISL_10969470, EPI_ISL_10969471, EPI_ISL_10969472, EPI_ISL_10969473, EPI_ISL_10969474, EPI_ISL_10969475, EPI_ISL_10969476, EPI_ISL_10969477, EPI_ISL_10969481, EPI_ISL_10969503, EPI_ISL_10969509, EPI_ISL_10969521, EPI_ISL_10969612, EPI_ISL_10969613, EPI_ISL_10969845, EPI_ISL_10970235, EPI_ISL_10970479, EPI_ISL_10977347, EPI_ISL_10977423, EPI_ISL_10977817, EPI_ISL_10978119, EPI_ISL_10978329, EPI_ISL_10978528, EPI_ISL_10979009, EPI_ISL_10979174

see above RSUP dr. Sardjito Genetics Working Group (Pokja Genetik) Faculty of Medicine, Public Health and Nursing Universitas Gadjah Mada (FK-KMK UGM);

Balai Besar Teknik Kesehatan Lingkungan Dan Pengendalian Penyakit (BBTKLPP) Yogyakarta; Department of Microbiology FK-KMK UGM; Laboratorium Diagnostik Yayasan Tahija World Mosquito Program (WMP) Yogyakarta Center for Tropical Medicine FK-KMK UGM; Integrated Research Center FK-KMK UGM; Department of Computer Science and Electronics FMIPA UGM; RSUP Dr. Sardjito

Afiahayati; Aning; Dwi AA Nugrahaningsih; Dyah A Puspitarani; Edita M Devana; Edwin W. Daniwijaya; Eggi Arguni; Endah Supriyati; Esensi T Geometri; Fadila D T Utami; Fatin; Gita C Gabriela; Gunadi; Hani; Havid; Hendra Wibawa; Ika Trisnawati; Irene; Khanza A Vujira; Kristy Iskandar; Lanang; Lanang Aditama; Laudria S Eryvinka; Ludhang P. Rizki; Mohamad S. Hakim; Nathania; Nathania C P Kinasih; Nungki Anggorowati; Rahmi Nanda; Riat E Khair; Siswanto; Sri Fatmawati; Stella; Titik Nuryastuti; Tri Wibawa; Verrell Christopher

EPI_ISL_9570723, EPI_ISL_9570724, EPI_ISL_9570726, EPI_ISL_9570728

RSUP dr. Sardjito Genetics Working Group (Pokja Genetik) Faculty of Medicine, Public Health and Nursing Universitas Gadjah Mada (FK-KMK UGM); Disease Investigation Center Wates Ministry of Agriculture Indonesia; Department of Microbiology FK-KMK UGM; Laboratorium Diagnostik Yayasan Tahija World Mosquito Program (WMP) Yogyakarta Center for Tropical Medicine FK-KMK UGM; Integrated

Research Center FK-KMK UGM; Department of Computer Science and Electronics FMIPA UGM; RSUP Dr. Sardjito

Afiahayati; Dwi AA Nugrahaningsih; Dyah A Puspitarani; Edwin W. Daniwijaya; Eggi Arguni; Endah Supriyati; Fadila D T Utami; Gita C Gabriela; Gunadi; Hendra Wibawa; Ika Trisnawati; Khanza A Vujira; Kristy Iskandar; Lanang Aditama; Laudria S Eryvinka; Ludhang P. Rizki; Mohamad S. Hakim; Nungki Anggorowati; Riat E Khair; Siswanto; Titik Nuryastuti; Tri Wibawa

EPI_ISL_4968082 Rumah Sakit Bhayangkara Polda DIY Genetics Working Group (Pokja Genetik) Faculty of Medicine, Public Health and Nursing Universitas Gadjah Mada (FK-KMK UGM);

Disease Investigation Center Wates Ministry of Agriculture Indonesia; Department of Microbiology FK-KMK UGM; Laboratorium Diagnostik Yayasan Tahija World Mosquito Program (WMP) Yogyakarta Center for Tropical Medicine FK-KMK UGM; Integrated Research Center FK-KMK UGM; Department of Computer Science and Electronics FMIPA UGM; RSUP Dr. Sardjito

Afiahayati; Dwi AA Nugrahaningsih; Dwi Indaryati; Edwin W. Daniwijaya; Eggi Arguni; Endah Supriyati; Gunadi; Hana F Hanifin; Hendra Wibawa; Indarto Sulistiyono; Irene Tania; Kristy Iskandar; Ludhang P. Rizki; Marcellus; Mohamad S. Hakim; Nungki Anggorowati; Pramesti G Dewi; Siswanto; Titik Nuryastuti; Tri Wibawa

EPI_ISL_10657577, EPI_ISL_10657727, EPI_ISL_10657728, EPI_ISL_10657729

Rumah Sakit JIH SOLO - The Ultimate Value Healthcare Genetics Working Group (Pokja Genetik) Faculty of Medicine, Public Health and Nursing Universitas Gadjah Mada (FK-KMK UGM);

Disease Investigation Center Wates Ministry of Agriculture Indonesia; Department of Microbiology FK-KMK UGM; Laboratorium Diagnostik Yayasan Tahija World Mosquito Program (WMP) Yogyakarta Center for Tropical Medicine FK-KMK UGM; Integrated Research Center FK-KMK UGM; Department of Computer Science and Electronics FMIPA UGM; RSUP Dr. Sardjito

Afiahayati; Dwi AA Nugrahaningsih; Dyah A Puspitarani; Edita M Devana; Edwin W. Daniwijaya; Eggi Arguni; Endah Supriyati; Esensi T Geometri; Fadila D T Utami; Gita C Gabriela; Gunadi; Hendra Wibawa; Ika Trisnawati; Khanza A Vujira; Kristy Iskandar; Ludhang P. Rizki; Mohamad S. Hakim; Nungki Anggorowati; Riat E Khair; Siswanto; Titik Nuryastuti; Tri Wibawa; Verrell Christopher

EPI_ISL_10658792 Rumah Sakit JIH Solo Genetics Working Group (Pokja Genetik) Faculty of Medicine, Public Health and Nursing Universitas Gadjah Mada (FK-KMK UGM);

Disease Investigation Center Wates Ministry of Agriculture Indonesia; Department of Microbiology FK-KMK UGM; Laboratorium Diagnostik Yayasan Tahija World Mosquito Program (WMP) Yogyakarta Center for Tropical Medicine FK-KMK UGM; Integrated Research Center FK-KMK UGM; Department of Computer Science and Electronics FMIPA UGM; RSUP Dr. Sardjito

Afiahayati; Dwi AA Nugrahaningsih; Dyah A Puspitarani; Edita M Devana; Edwin W. Daniwijaya; Eggi Arguni; Endah Supriyati; Esensi T Geometri; Fadila D T Utami; Gita C Gabriela; Gunadi; Hendra Wibawa; Ika Trisnawati; Khanza A Vujira; Kristy Iskandar; Ludhang P. Rizki; Mohamad S. Hakim; Nungki Anggorowati; Riat E Khair; Siswanto; Titik Nuryastuti; Tri Wibawa; Verrell Christopher

EPI_ISL_10657579, EPI_ISL_10657731, EPI_ISL_10658770

EPI_ISL_9487326, EPI_ISL_9487383, EPI_ISL_9487411

Rumah Sakit Kasih Ibu Surakarta Genetics Working Group (Pokja Genetik) Faculty of Medicine, Public Health and Nursing Universitas Gadjah Mada (FK-KMK UGM); Disease Investigation Center Wates Ministry of Agriculture Indonesia; Department of Microbiology FK-KMK UGM; Laboratorium Diagnostik Yayasan Tahija World Mosquito Program (WMP) Yogyakarta Center for Tropical Medicine FK-KMK UGM; Integrated

Research Center FK-KMK UGM; Department of Computer Science and Electronics FMIPA UGM; RSUP Dr. Sardjito

UPTD Labkes Kab. Kebumen Genetics Working Group (Pokja Genetik) Faculty of Medicine, Public Health and Nursing Universitas Gadjah Mada (FK-KMK UGM); Disease Investigation Center Wates Ministry of Agriculture Indonesia; Department of Microbiology FK-KMK UGM; Laboratorium Diagnostik Yayasan Tahija World Mosquito Program (WMP) Yogyakarta Center for Tropical Medicine FK-KMK UGM; Integrated

Research Center FK-KMK UGM; Department of Computer Science and Electronics FMIPA UGM; RSUP Dr. Sardjito

Afiahayati; Dwi AA Nugrahaningsih; Dyah A Puspitarani; Edita M Devana; Edwin W. Daniwijaya; Eggi Arguni; Endah Supriyati; Esensi T Geometri; Fadila D T Utami; Gita C Gabriela; Gunadi; Hendra Wibawa; Ika Trisnawati; Khanza A Vujira; Kristy Iskandar; Ludhang P. Rizki; Mohamad S. Hakim; Nungki Anggorowati; Riat E Khair; Siswanto; Titik Nuryastuti; Tri Wibawa; Verrell Christopher

Afiahayati; Dwi AA Nugrahaningsih; Dyah A Puspitarani; Edwin W. Daniwijaya; Eggi Arguni; Endah Supriyati; Fadila D T Utami; Gita C Gabriela; Gunadi; Hendra Wibawa; Ika Trisnawati; Khanza A Vujira; Kristy Iskandar; Lanang Aditama; Laudria S Eryvinka; Ludhang P. Rizki; Mohamad S. Hakim; Nungki Anggorowati; Riat E Khair; Siswanto; Titik Nuryastuti; Tri Wibawa

# We gratefully acknowledge the following Authors from the Originating laboratories responsible for obtaining the specimens, as well as the Submitting laboratories where the genome data were generated and shared via GISAID, on which this research is based.

All Submitters of data may be contacted directly via [www.gisaid.org](http://www.gisaid.org/) Authors are sorted alphabetically.

## Accession ID Originating Laboratory Submitting Laboratory Authors

EPI_ISL_2943235 BBTKLPP Yogyakarta Genetics Working Group (Pokja Genetik) Faculty of Medicine, Public Health and Nursing Universitas Gadjah Mada (FK-KMK UGM); Disease

Investigation Center Wates Ministry of Agriculture Indonesia; Department of Microbiology FK-KMK UGM; Laboratorium Diagnostik Yayasan Tahija World Mosquito Program (WMP) Yogyakarta Center for Tropical Medicine FK-KMK UGM; Integrated Research Center FK-KMK UGM; Department of Computer Science and Electronics FMIPA UGM; RSUP Dr. Sardjito

Afiahayati; Dwi AA Nugrahaningsih; Dwi Indaryati; Edwin W. Daniwijaya; Eggi Arguni; Endah Supriyati; Gunadi; Hana F Hanifin; Hendra Wibawa; Indarto Sulistiyono; Irene Tania; Kristy Iskandar; Ludhang P. Rizki; Marcellus; Mohamad S. Hakim; Nungki Anggorowati; Pramesti G Dewi; Siswanto; Titik Nuryastuti; Tri Wibawa

EPI_ISL_2964704, EPI_ISL_2964706

EPI_ISL_2964697, EPI_ISL_2964700

DINKES KAB. GROBOGAN Genetics Working Group (Pokja Genetik) Faculty of Medicine, Public Health and Nursing Universitas Gadjah Mada (FK-KMK UGM); Disease Investigation Center Wates Ministry of Agriculture Indonesia; Department of Microbiology FK-KMK UGM; Laboratorium Diagnostik Yayasan Tahija World Mosquito Program (WMP) Yogyakarta Center for Tropical Medicine FK-KMK UGM; Integrated Research Center FK-KMK UGM; Department of

Computer Science and Electronics FMIPA UGM; RSUP Dr. Sardjito

DINKES KAB. JEPARA Genetics Working Group (Pokja Genetik) Faculty of Medicine, Public Health and Nursing Universitas Gadjah Mada (FK-KMK UGM); Disease Investigation Center Wates Ministry of Agriculture Indonesia; Department of Microbiology FK-KMK UGM; Laboratorium Diagnostik Yayasan Tahija World Mosquito Program (WMP) Yogyakarta Center for Tropical Medicine FK-KMK UGM; Integrated Research Center FK-KMK UGM; Department of

Computer Science and Electronics FMIPA UGM; RSUP Dr. Sardjito

Afiahayati; Dwi AA Nugrahaningsih; Dwi Indaryati; Dyah A Puspitarani; Edwin W. Daniwijaya; Eggi Arguni; Endah Supriyati; Gunadi; Hana F Hanifin; Hendra Wibawa; Indarto Sulistiyono; Irene Tania; Kristy Iskandar; Ludhang P. Rizki; Marcellus; Mohamad S. Hakim; Nungki Anggorowati; Pramesti G Dewi; Siswanto; Susan Simanjaya; Titik Nuryastuti; Tri Wibawa

Afiahayati; Alvina A Setiawan; Cita S Amalia; Dwi AA Nugrahaningsih; Dwi Indaryati; Edwin W. Daniwijaya; Eggi Arguni; Endah Supriyati; Gunadi; Hendra Wibawa; Indarto Sulistiyono; Khanza a Vujira; Kristy Iskandar; Ludhang P. Rizki; Marcellus; Mohamad S. Hakim; Nungki Anggorowati; Pramesti G Dewi; Siswanto; Titik Nuryastuti; Tri Wibawa

EPI_ISL_2954684 DKK Gunungkidul/ Puskesmas Girisubo Genetics Working Group (Pokja Genetik) Faculty of Medicine, Public Health and Nursing Universitas Gadjah Mada (FK-KMK UGM); Disease

Investigation Center Wates Ministry of Agriculture Indonesia; Department of Microbiology FK-KMK UGM; Laboratorium Diagnostik Yayasan Tahija World Mosquito Program (WMP) Yogyakarta Center for Tropical Medicine FK-KMK UGM; Integrated Research Center FK-KMK UGM; Department of Computer Science and Electronics FMIPA UGM; RSUP Dr. Sardjito

Afiahayati; Alvina A Setiawan; Cita S Amalia; Dwi AA Nugrahaningsih; Dwi Indaryati; Edwin W. Daniwijaya; Eggi Arguni; Endah Supriyati; Gunadi; Hendra Wibawa; Indarto Sulistiyono; Kristy Iskandar; Ludhang P. Rizki; Marcellus; Mohamad S. Hakim; Nungki Anggorowati; Pramesti G Dewi; Siswanto; Titik Nuryastuti; Tri Wibawa

EPI_ISL_2955335, EPI_ISL_2955586, EPI_ISL_2955597, EPI_ISL_2955598

DKK Gunungkidul/ Puskesmas Karangmojo I Genetics Working Group (Pokja Genetik) Faculty of Medicine, Public Health and Nursing Universitas Gadjah Mada (FK-KMK UGM); Disease Investigation Center Wates Ministry of Agriculture Indonesia; Department of Microbiology FK-KMK UGM; Laboratorium Diagnostik Yayasan Tahija World Mosquito Program (WMP) Yogyakarta Center for Tropical Medicine FK-KMK UGM; Integrated Research Center FK-KMK UGM; Department of

Computer Science and Electronics FMIPA UGM; RSUP Dr. Sardjito

Afiahayati; Alvina A Setiawan; Dwi AA Nugrahaningsih; Dwi Indaryati; Dyah A Puspitarani; Edwin W. Daniwijaya; Eggi Arguni; Endah Supriyati; Gunadi; Hana F Hanifin; Hendra Wibawa; Indarto Sulistiyono; Irene Tania; Khanza a Vujira; Kristy Iskandar; Ludhang P. Rizki; Marcellus; Mohamad S. Hakim; Nungki Anggorowati; Pramesti G Dewi; Siswanto; Susan Simanjaya; Titik Nuryastuti; Tri Wibawa

EPI_ISL_2933110 DKK Sleman/Puskesmas Depok 1 Genetics Working Group (Pokja Genetik) Faculty of Medicine, Public Health and Nursing Universitas Gadjah Mada (FK-KMK UGM); Disease

Investigation Center Wates Ministry of Agriculture Indonesia; Department of Microbiology FK-KMK UGM; Laboratorium Diagnostik Yayasan Tahija World Mosquito Program (WMP) Yogyakarta Center for Tropical Medicine FK-KMK UGM; Integrated Research Center FK-KMK UGM; Department of Computer Science and

Afiahayati; Alvina A Setiawan; Dwi AA Nugrahaningsih; Dwi Indaryati; Edwin W. Daniwijaya; Eggi Arguni; Endah Supriyati; Gunadi; Hendra Wibawa; Indarto Sulistiyono; Khanza a Vujira; Kristy Iskandar; Ludhang P. Rizki; Marcellus; Mohamad S. Hakim; Nungki Anggorowati; Pramesti G Dewi; Siswanto; Titik Nuryastuti; Tri Wibawa

EPI_ISL_2932613, EPI_ISL_2933109, EPI_ISL_2943237, EPI_ISL_2955609

EPI_ISL_2955627, EPI_ISL_2955628

DKK Sleman/Puskesmas Depok 1 Genetics Working Group (Pokja Genetik) Faculty of Medicine, Public Health and Nursing Universitas Gadjah Mada (FK-KMK UGM); Disease Investigation Center Wates Ministry of Agriculture Indonesia; Department of Microbiology FK-KMK UGM; Laboratorium Diagnostik Yayasan Tahija World Mosquito Program (WMP) Yogyakarta Center for Tropical Medicine FK-KMK UGM; Integrated Research Center FK-KMK UGM; Department of

Computer Science and Electronics FMIPA UGM; RSUP Dr. Sardjito

DKK Sleman/Puskesmas Ngemplak 2 Genetics Working Group (Pokja Genetik) Faculty of Medicine, Public Health and Nursing Universitas Gadjah Mada (FK-KMK UGM); Disease Investigation Center Wates Ministry of Agriculture Indonesia; Department of Microbiology FK-KMK UGM; Laboratorium Diagnostik Yayasan Tahija World Mosquito Program (WMP) Yogyakarta Center for Tropical Medicine FK-KMK UGM; Integrated Research Center FK-KMK UGM; Department of

Computer Science and Electronics FMIPA UGM; RSUP Dr. Sardjito

Afiahayati; Alvina A Setiawan; Cita S Amalia; Dwi AA Nugrahaningsih; Dwi Indaryati; Dyah A Puspitarani; Edwin W. Daniwijaya; Eggi Arguni; Endah Supriyati; Gunadi; Hana F Hanifin; Hendra Wibawa; Indarto Sulistiyono; Irene Tania; Kristy Iskandar; Ludhang P. Rizki; Marcellus; Mohamad S. Hakim; Nungki Anggorowati; Pramesti G Dewi; Siswanto; Susan Simanjaya; Titik Nuryastuti; Tri Wibawa

Afiahayati; Dwi AA Nugrahaningsih; Dwi Indaryati; Dyah A Puspitarani; Edwin W. Daniwijaya; Eggi Arguni; Endah Supriyati; Gunadi; Hana F Hanifin; Hendra Wibawa; Indarto Sulistiyono; Irene Tania; Kristy Iskandar; Ludhang P. Rizki; Marcellus; Mohamad S. Hakim; Nungki Anggorowati; Pramesti G Dewi; Siswanto; Susan Simanjaya; Titik Nuryastuti; Tri Wibawa

EPI_ISL_2932610 KOTA Yogya DKK Gondokusuman II Genetics Working Group (Pokja Genetik) Faculty of Medicine, Public Health and Nursing Universitas Gadjah Mada (FK-KMK UGM); Disease

Investigation Center Wates Ministry of Agriculture Indonesia; Department of Microbiology FK-KMK UGM; Laboratorium Diagnostik Yayasan Tahija World Mosquito Program (WMP) Yogyakarta Center for

EPI_ISL_2964681 Puskesmas Colomadu 1 DKK Karanganyar Genetics Working Group (Pokja Genetik) Faculty of Medicine, Public Health and Nursing Universitas Gadjah Mada (FK-KMK UGM); Disease

Investigation Center Wates Ministry of Agriculture Indonesia; Department of Microbiology FK-KMK UGM; Laboratorium Diagnostik Yayasan Tahija World Mosquito Program (WMP) Yogyakarta Center for Tropical Medicine FK-KMK UGM; Integrated Research Center FK-KMK UGM; Department of Computer Science and Electronics FMIPA UGM; RSUP Dr. Sardjito

Afiahayati; Alvina A Setiawan; Dwi AA Nugrahaningsih; Dwi Indaryati; Edwin W. Daniwijaya; Eggi Arguni; Endah Supriyati; Gunadi; Hendra Wibawa; Indarto Sulistiyono; Khanza a Vujira; Kristy Iskandar; Ludhang P. Rizki; Marcellus; Mohamad S. Hakim; Nungki Anggorowati; Pramesti G Dewi; Siswanto; Titik Nuryastuti; Tri Wibawa

Afiahayati; Alvina A Setiawan; Dwi AA Nugrahaningsih; Dwi Indaryati; Edwin W. Daniwijaya; Eggi Arguni; Endah Supriyati; Gunadi; Hendra Wibawa; Indarto Sulistiyono; Khanza a Vujira; Kristy Iskandar; Ludhang P. Rizki; Marcellus; Mohamad S. Hakim; Nungki Anggorowati; Pramesti G Dewi; Siswanto; Titik Nuryastuti; Tri Wibawa

EPI_ISL_2964917, EPI_ISL_2964918, EPI_ISL_2964919, EPI_ISL_2964920, EPI_ISL_2964921

EPI_ISL_2964707, EPI_ISL_2964872

RS Dr. OEN KANDANG SAPI SOLO Genetics Working Group (Pokja Genetik) Faculty of Medicine, Public Health and Nursing Universitas Gadjah Mada (FK-KMK UGM); Disease Investigation Center Wates Ministry of Agriculture Indonesia; Department of Microbiology FK-KMK UGM; Laboratorium Diagnostik Yayasan Tahija World Mosquito Program (WMP) Yogyakarta Center for Tropical Medicine FK-KMK UGM; Integrated Research Center FK-KMK UGM; Department of

Computer Science and Electronics FMIPA UGM; RSUP Dr. Sardjito

RS Dr. Oen Kandang Sapi Solo Genetics Working Group (Pokja Genetik) Faculty of Medicine, Public Health and Nursing Universitas Gadjah Mada (FK-KMK UGM); Disease Investigation Center Wates Ministry of Agriculture Indonesia; Department of Microbiology FK-KMK UGM; Laboratorium Diagnostik Yayasan Tahija World Mosquito Program (WMP) Yogyakarta Center for Tropical Medicine FK-KMK UGM; Integrated Research Center FK-KMK UGM; Department of

Computer Science and Electronics FMIPA UGM; RSUP Dr. Sardjito

Afiahayati; Alvina A Setiawan; Cita S Amalia; Dwi AA Nugrahaningsih; Dwi Indaryati; Dyah A Puspitarani; Edwin W. Daniwijaya; Eggi Arguni; Endah Supriyati; Gunadi; Hana F Hanifin; Hendra Wibawa; Indarto Sulistiyono; Irene Tania; Khanza a Vujira; Kristy Iskandar; Ludhang P. Rizki; Marcellus; Mohamad S. Hakim; Nungki Anggorowati; Pramesti G Dewi; Siswanto; Susan Simanjaya; Titik Nuryastuti; Tri Wibawa

Afiahayati; Alvina A Setiawan; Cita S Amalia; Dwi AA Nugrahaningsih; Dwi Indaryati; Dyah A Puspitarani; Edwin W. Daniwijaya; Eggi Arguni; Endah Supriyati; Gunadi; Hendra Wibawa; Indarto Sulistiyono; Kristy Iskandar; Ludhang P. Rizki; Marcellus; Mohamad S. Hakim; Nungki Anggorowati; Pramesti G Dewi; Siswanto; Susan Simanjaya; Titik Nuryastuti; Tri Wibawa

EPI_ISL_2955608 RSA UGM Genetics Working Group (Pokja Genetik) Faculty of Medicine, Public Health and Nursing Universitas Gadjah Mada (FK-KMK UGM); Disease

Investigation Center Wates Ministry of Agriculture Indonesia; Department of Microbiology FK-KMK UGM; Laboratorium Diagnostik Yayasan Tahija World Mosquito Program (WMP) Yogyakarta Center for Tropical Medicine FK-KMK UGM; Integrated Research Center FK-KMK UGM; Department of Computer Science and Electronics FMIPA UGM; RSUP Dr. Sardjito

EPI_ISL_2964684, EPI_ISL_2964688, EPI_ISL_2964692, EPI_ISL_2964941, EPI_ISL_2964942, EPI_ISL_2964943, EPI_ISL_2964944, EPI_ISL_2964956, EPI_ISL_2964957

see above RSUD DR. MOEWARDI Genetics Working Group (Pokja Genetik) Faculty of Medicine, Public Health and Nursing Universitas Gadjah Mada (FK-KMK UGM); Disease

Investigation Center Wates Ministry of Agriculture Indonesia; Department of Microbiology FK-KMK UGM; Laboratorium Diagnostik Yayasan Tahija World Mosquito Program (WMP) Yogyakarta Center for Tropical Medicine FK-KMK UGM; Integrated Research Center FK-KMK UGM; Department of Computer Science and Electronics FMIPA UGM; RSUP Dr. Sardjito

EPI_ISL_2964677 RSUD DR. MOEWARDI Surakarta Genetics Working Group (Pokja Genetik) Faculty of Medicine, Public Health and Nursing Universitas Gadjah Mada (FK-KMK UGM); Disease

Investigation Center Wates Ministry of Agriculture Indonesia; Department of Microbiology FK-KMK UGM; Laboratorium Diagnostik Yayasan Tahija World Mosquito Program (WMP) Yogyakarta Center for Tropical Medicine FK-KMK UGM; Integrated Research Center FK-KMK UGM; Department of Computer Science and Electronics FMIPA UGM; RSUP Dr. Sardjito

Afiahayati; Dwi AA Nugrahaningsih; Dwi Indaryati; Dyah A Puspitarani; Edwin W. Daniwijaya; Eggi Arguni; Endah Supriyati; Gunadi; Hendra Wibawa; Indarto Sulistiyono; Kristy Iskandar; Ludhang P. Rizki; Marcellus; Mohamad S. Hakim; Nungki Anggorowati; Pramesti G Dewi; Siswanto; Susan Simanjaya; Titik Nuryastuti; Tri Wibawa

Afiahayati; Alvina A Setiawan; Cita S Amalia; Dwi AA Nugrahaningsih; Dwi Indaryati; Dyah A Puspitarani; Edwin W. Daniwijaya; Eggi Arguni; Endah Supriyati; Gunadi; Hana F Hanifin; Hendra Wibawa; I Putu Aditio A; Indarto Sulistiyono; Irene Tania; Kristy Iskandar; Ludhang P. Rizki; Marcellus; Mohamad S. Hakim; Nungki Anggorowati; Pramesti G Dewi; Siswanto; Susan Simanjaya; Titik Nuryastuti; Tri Wibawa

Afiahayati; Alvina A Setiawan; Dwi AA Nugrahaningsih; Dwi Indaryati; Edwin W. Daniwijaya; Eggi Arguni; Endah Supriyati; Gunadi; Hendra Wibawa; Indarto Sulistiyono; Khanza a Vujira; Kristy Iskandar; Ludhang P. Rizki; Marcellus; Mohamad S. Hakim; Nungki Anggorowati; Pramesti G Dewi; Siswanto; Titik Nuryastuti; Tri Wibawa

EPI_ISL_2954064 RSUD Panembahan Senopati, Bantul Genetics Working Group (Pokja Genetik) Faculty of Medicine, Public Health and Nursing Universitas Gadjah Mada (FK-KMK UGM); Disease

Investigation Center Wates Ministry of Agriculture Indonesia; Department of Microbiology FK-KMK UGM; Laboratorium Diagnostik Yayasan Tahija World Mosquito Program (WMP) Yogyakarta Center for Tropical Medicine FK-KMK UGM; Integrated Research Center FK-KMK UGM; Department of Computer

Afiahayati; Dwi AA Nugrahaningsih; Dwi Indaryati; Edwin W. Daniwijaya; Eggi Arguni; Endah Supriyati; Gunadi; Hana F Hanifin; Hendra Wibawa; Indarto Sulistiyono; Irene Tania; Kristy Iskandar; Ludhang P. Rizki; Marcellus; Mohamad S. Hakim; Nungki Anggorowati; Pramesti G Dewi; Siswanto; Titik Nuryastuti; Tri Wibawa

EPI_ISL_2502616, EPI_ISL_2502627, EPI_ISL_2502628, EPI_ISL_2502629, EPI_ISL_2502667, EPI_ISL_2502687, EPI_ISL_2502717, EPI_ISL_2502763, EPI_ISL_2502764, EPI_ISL_2502765, EPI_ISL_2506039, EPI_ISL_2506040, EPI_ISL_2506041, EPI_ISL_2534078, EPI_ISL_2534079, EPI_ISL_2534082, EPI_ISL_2534289, EPI_ISL_2534338, EPI_ISL_2534346, EPI_ISL_2534376, EPI_ISL_2534377, EPI_ISL_2534379, EPI_ISL_2534454, EPI_ISL_2534456, EPI_ISL_2534457, EPI_ISL_2534458, EPI_ISL_2534490, EPI_ISL_2534518

see above RSUD dr. LOEKMONO HADI Genetics Working Group (Pokja Genetik) Faculty of Medicine, Public Health and Nursing Universitas Gadjah Mada (FK-KMK UGM); Disease

Investigation Center Wates Ministry of Agriculture Indonesia; Department of Microbiology FK-KMK UGM; Laboratorium Diagnostik Yayasan Tahija World Mosquito Program (WMP) Yogyakarta Center for Tropical Medicine FK-KMK UGM; Integrated Research Center FK-KMK UGM; Department of Computer Science and Electronics FMIPA UGM; RSUP Dr. Sardjito

EPI_ISL_2955607 RSUP Dr Sardjito Genetics Working Group (Pokja Genetik) Faculty of Medicine, Public Health and Nursing Universitas Gadjah Mada (FK-KMK UGM); Disease

Investigation Center Wates Ministry of Agriculture Indonesia; Department of Microbiology FK-KMK UGM; Laboratorium Diagnostik Yayasan Tahija World Mosquito Program (WMP) Yogyakarta Center for Tropical Medicine FK-KMK UGM; Integrated Research Center FK-KMK UGM; Department of Computer Science and Electronics FMIPA UGM; RSUP Dr. Sardjito

EPI_ISL_2943173 RSUP Dr. Sardjito Genetics Working Group (Pokja Genetik) Faculty of Medicine, Public Health and Nursing Universitas Gadjah Mada (FK-KMK UGM); Disease

Investigation Center Wates Ministry of Agriculture Indonesia; Department of Microbiology FK-KMK UGM; Laboratorium Diagnostik Yayasan Tahija World Mosquito Program

EPI_ISL_2955606 RSUP Sardjito Genetics Working Group (Pokja Genetik) Faculty of Medicine, Public Health and Nursing Universitas Gadjah Mada (FK-KMK UGM); Disease

Investigation Center Wates Ministry of Agriculture Indonesia; Department of Microbiology FK-KMK UGM; Laboratorium Diagnostik Yayasan Tahija World Mosquito Program (WMP) Yogyakarta Center for Tropical Medicine FK-KMK UGM; Integrated Research Center FK-KMK UGM; Department of Computer Science and Electronics FMIPA UGM; RSUP Dr. Sardjito

Afiahayati; Alvina A Setiawan; Cita S Amalia; Dwi AA Nugrahaningsih; Dwi Indaryati; Dyah A Puspitarani; Edwin W. Daniwijaya; Eggi Arguni; Endah Supriyati; Gunadi; Hana F Hanifin; Hendra Wibawa; Indarto Sulistiyono; Irene Tania; Khanza a Vujira; Kristy Iskandar; Ludhang P. Rizki; Marcellus; Mohamad S. Hakim; Nungki Anggorowati; Pramesti G Dewi; Siswanto; Susan Simanjaya; Titik Nuryastuti; Tri Wibawa

. Marcellus; Afiahayati; Bambang Sigit Riyanto; Dwi AA Nugrahaningsih; Edwin W. Daniwijaya; Eggi Arguni; Eko Budiono; Endah Supriyati; Gunadi; Hana F Hanifin; Hendra Wibawa; Heni Retnowulan; Ika Trisnawati; Ira Puspitawati; Irene Tania; Kristy Iskandar; Ludhang P. Rizki; Mohamad S. Hakim; Munawar Gani; Nungki Anggorowati; Nur Imma Fatimah Harahap; Nur Rahmi Ananda; Osman Sianipar; Riat El Khair; Satria Maulana; Siswanto; Sumardi; Titik Nuryastuti; Tri Wibawa; Umi Solekhah Intansari; Yunika Puspadewi; Elizabeth Henny Herningtiyas

. Marcellus; Afiahayati; Bambang Sigit Riyanto; Dwi AA Nugrahaningsih; Edwin W. Daniwijaya; Eggi Arguni; Eko Budiono; Endah Supriyati; Gunadi; Hana F Hanifin; Hendra Wibawa; Heni Retnowulan; Ika Trisnawati; Ira Puspitawati; Irene Tania; Kristy Iskandar; Ludhang P. Rizki; Mohamad S. Hakim; Munawar Gani; Nungki Anggorowati; Nur Imma Fatimah Harahap; Nur Rahmi Ananda; Osman Sianipar; Riat El Khair; Satria Maulana; Siswanto; Sumardi; Titik Nuryastuti; Tri Wibawa; Umi Solekhah Intansari; Yunika Puspadewi; Elizabeth Henny Herningtiyas

. Marcellus; Afiahayati; Alvina A Setiawan; Bambang Sigit Riyanto; Dwi AA Nugrahaningsih; Edwin W. Daniwijaya; Eggi Arguni; Eko Budiono; Endah Supriyati; Gunadi; Hendra Wibawa; Heni Retnowulan; Ika Trisnawati; Ira Puspitawati; Khanza a Vujira; Kristy Iskandar; Ludhang P. Rizki; Mohamad S. Hakim; Munawar Gani; Nungki Anggorowati; Nur Imma Fatimah Harahap; Nur Rahmi Ananda; Osman Sianipar; Riat El Khair; Satria Maulana; Siswanto; Sumardi; Titik Nuryastuti; Tri Wibawa; Umi Solekhah Intansari; Yunika Puspadewi; Elizabeth Henny Herningtiyas
